# Supplementary material for: Developing a Complex Understanding of Physical Activity in Cardiometabolic Disease from Low-to-Middle-Income Countries—A Qualitative Systematic Review with Meta-Synthesis
Source: Int J Environ Res Public Health. 2021 Nov 15;18(22):11977. doi: 10.3390/ijerph182211977 (PMC8619369; doi:10.3390/ijerph182211977)
Supplement: Supplementary file 1 [file ijerph-18-11977-s001.zip › File S1-Search strategy.pdf]

Search Strategies

PubMed 2
Scopus 5
Web of Science 7
CINAHL, AfricaWide, and SPORTdiscus (EBSCOhost) 13

PubMed

#1 "Qualitative Research"[Mesh] OR "Focus Groups"[Mesh] OR "Interview" [Publication Type] OR "Interviews as Topic"[Mesh] OR "Narration"[Mesh] OR "Personal Narratives as Topic"[Mesh] OR "Grounded Theory"[Mesh] OR "Tape Recording"[Mesh] OR thematic analys*[tiab] OR content analys*[tiab] OR focus group*[tiab] OR ethnograph*[tiab] OR ethnograf*[tiab] OR etnograf*[tiab] OR field stud*[tiab] OR phenomenolog*[tiab] OR narration*[tiab] OR narrative[tiab] OR qualitative stud*[tiab] OR qualitative analys*[tiab] OR qualitative research*[tiab] OR qualitative method*[tiab] OR multimethodolog*[tiab] OR mixed method*[tiab] OR grounded theory[tiab] OR audio recording*[tiab] OR tape recording*[tiab] OR audiotape*[tiab] OR ((semi-structured[tiab] OR semistructured[tiab] OR unstructured[tiab] OR informal[tiab] OR in-depth[tiab] OR indepth[tiab] OR face-to-face[tiab] OR structured[tiab] OR guide*[tiab]) AND (interview*[tiab] OR discussion*[tiab]))

#2 "insulin resistance"[MeSH] OR (insulin[tiab] AND (resistancy[tiab] OR resistance[tiab] OR resistant[tiab] OR sensitivity[tiab]))

#3 "Diabetes Mellitus, Type 2"[Mesh] OR ((diabetes[tiab] OR diabetic*[tiab]) AND (non insulin depend*[tiab] OR noninsulin depend*[tiab] OR noninsulindepend*[tiab] OR non insulindepend*[tiab] OR maturity onset*[tiab] OR adult onset*[tiab] OR slow onset*[tiab])) OR dm2[tiab] OR niddm[tiab] OR dm 2[tiab] OR t2d*[tiab] OR dm type 2[tiab] OR type 2 diabet*[tiab] OR dm type II[tiab] OR type two diabet*[tiab] OR type II diabet*[tiab] OR dm type II[tiab]

#4 "Metabolic Syndrome"[Mesh] OR "Glucose Intolerance"[Mesh] OR "Blood Glucose"[Mesh] OR "Hyperglycemia"[Mesh] OR "Glycated Hemoglobin A"[Mesh] OR "Diabetes Mellitus"[Mesh:NoExp] OR "Diabetes Mellitus, Type 2"[Mesh] OR "Prediabetic State"[Mesh] OR "Insulin Resistance"[Mesh] OR "Hyperinsulinism"[Mesh] OR "Blood Pressure"[Mesh] OR "Hypertension"[Mesh] OR "Arterial Pressure"[Mesh] OR "Triglycerides"[Mesh] OR "Cholesterol"[Mesh] OR "Lipoproteins, HDL"[Mesh] OR "Lipoproteins, LDL"[Mesh] OR "Lipoproteins, VLDL"[Mesh] OR "Lipoproteins, IDL"[Mesh] OR "Obesity, Abdominal"[Mesh] OR "Body Weight Changes"[Mesh] OR "Body Weights and Measures"[Mesh] OR "Dyslipidemias"[Mesh] OR "Waist Circumference"[Mesh] OR "Waist-Hip Ratio"[Mesh] OR "metabolic syndrome"[tiab] OR "glucose intoleran*"[tiab] OR "glucose toleran*"[tiab] OR "glucose dysregulation"[tiab] OR "igt"[tiab] OR "blood glucose"[tiab] OR "blood sugar"[tiab] OR "plasma glucose"[tiab] OR "glucose level*"[tiab] OR "glucose blood"[tiab] OR "fasting glucose"[tiab] OR "hyperglycem*"[tiab] OR "hyperglycaem*"[tiab] OR "Hb A1c"[tiab] OR "HbA1c"[tiab] OR "Hemoglobin A1c"[tiab] OR "diabet*"[tiab] OR "type 2 dm"[tiab] OR "type II dm"[tiab] OR "dm type 2"[tiab] OR "dm type II"[tiab] OR "NIDDM"[tiab] OR "IDDM"[tiab] OR "insulin resistan*"[tiab] OR "insulin sensitiv*"[tiab] OR "insulin insensitiv*"[tiab] OR "homa-ir"[tiab] OR "homa2"[tiab] OR "hyperinsulin*"[tiab] OR "prediabet*"[tiab] OR "blood pressure"[tiab] OR "diastolic pressure"[tiab] OR "systolic pressure"[tiab] OR "pulse pressure"[tiab] OR "arterial pressure"[tiab] OR "aortic pressure"[tiab] OR "hypertensi*"[tiab] OR "triglyceride*"[tiab] OR "triacylglycerol*"[tiab] OR "triacetylglycerol"[tiab] OR "triacetyl-glycerol"[tiab] OR "trielaidin"[tiab] OR "trioleoylglycerol"[tiab] OR "trioleyl glycerol"[tiab] OR "glycerol trioleate"[tiab] OR "trioleate-glycerin"[tiab] OR "hypertriglyceridemi*"[tiab] OR "cholesterol"[tiab] OR "hdl"[tiab] OR "ldl"[tiab] OR "vldl"[tiab] OR "idl"[tiab] OR "density lipoprotein*"[tiab] OR "hdl lipoprotein*"[tiab] OR

"low density lipoprotein*"[tiab] OR "high density lipoprotein*"[tiab] OR "very low lipoprotein*"[tiab] OR "obesit*"[tiab] OR "obese*"[tiab] OR "body mass index"[tiab] OR "bmi"[tiab] OR "body weight"[tiab] OR "body fat*"[tiab] OR "adiposit*"[tiab] OR "waist hip ratio*"[tiab] OR "waist to hip*"[tiab] OR "waist hip ratio*"[tiab] OR "waist circumference*"[tiab] OR "weight change*"[tiab] OR "weight gain*"[tiab] OR "weight loss*"[tiab] OR "weight reduction*"[tiab] OR "dyslipem*"[tiab] OR "dyslipidem*"[tiab] OR "dyslipaem*"[tiab] OR "dyslipidaem*"[tiab] OR "dyslipoprotein*"[tiab] OR "hyperlipemi*"[tiab] OR "hyperlipid*"[tiab] OR "lipidemi*"[tiab] OR "lipidaemi*"[tiab] OR "lipemi*"[tiab] OR "hypercholesterolemi*"[tiab] OR "hypercholesteremi*"[tiab] OR "hyperlipoproteinemi*"[tiab] OR "hypoprebetalipoproteinemi*"[tiab]

#5 "Cardiovascular Diseases"[Mesh] OR "Hyperlipidemias"[Mesh] OR "Cholesterol"[Mesh] OR "Stroke"[Mesh] OR cardiovascular disease*[tiab] OR cvd[tiab] OR coronary disease*[tiab] OR heart disease*[tiab] OR atherosclerosis[tiab] OR arteriosclerosis[tiab] OR hypertension[tiab] OR blood pressure[tiab] OR cholesterol[tiab] OR stroke*[tiab] OR peripheral vascular disease*[tiab] OR peripheral arterial disease*[tiab] OR hypercholesterol*[tiab] OR hyperlipid*[tiab]

#6 south east asia*[tw] OR middle east*[tw] OR Afghan*[tw] OR Angola*[tw] OR Algeria*[tw] OR Albania*[tw] OR Armenia*[tw] OR Argentina*[tw] OR Azerbaijan*[tw] OR Bangladesh*[tw] OR Belarus*[tw] OR Benin*[tw] OR Bhutan*[tw] OR birma*[tw] OR Boliv*[tw] OR Botswan*[tw] OR Bosnia*[tw] OR Brazil*[tw] OR Bulgaria*[tw] OR Burkina Faso*[tw] OR Burundi*[tw] OR Cabo Verde*[tw] OR Cambod*[tw] OR Cameroon*[tw] OR Cape Verd*[tw] OR Central Africa*[tw] OR Chad*[tw] OR China*[tw] OR Chinese*[tw] OR Colombia*[tw] OR Comoro*[tw] OR Congo*[tw] OR Costa Rica*[tw] OR Cote d'Ivoire*[tw] OR Cuba*[tw] OR Dominica*[tw] OR Djibouti*[tw] OR East Africa*[tw] OR Eastern Africa*[tw] OR Ecuador*[tw] OR Egypt*[tw] OR El Salvador*[tw] OR Equatorial Guinea*[tw] OR Eritre*[tw] OR Ethiopia*[tw] OR Gabon*[tw] OR Gambia*[tw] OR Gaza*[tw] OR Ghan*[tw] OR Georgia*[tw] OR Grenada*[tw] OR Guatemal*[tw] OR Guinea*[tw] OR Guyana*[tw] OR Haiti*[tw] OR Hondur*[tw] OR India*[tw] OR Indones*[tw] OR Iran*[tw] OR Iraq*[tw] OR Jamaica*[tw] OR Jordan*[tw] OR Ivory Coast*[tw] OR Kazakhstan*[tw] OR Kenya*[tw] OR Kiribati*[tw] OR Kosovo*[tw] OR Kyrgyz*[tw] OR Lao PDR*[tw] OR Lesotho*[tw] OR Lebanon*[tw] OR Libya*[tw] OR Liberia*[tw] OR Macedonia*[tw] OR Madagascar*[tw] OR Malaw*[tw] OR Mali[tw] OR Mauritan*[tw] OR Malaysia*[tw] OR Maldive*[tw] OR Marshall*[tw] OR Micronesi*[tw] OR Mexic*[tw] OR Moldov*[tw] OR Mongolia*[tw] OR Montenegr*[tw] OR Morocc*[tw] OR Mozambiqu*[tw] OR Myanmar*[tw] OR Namibia*[tw] OR Nepal*[tw] OR Nicaragua*[tw] OR Niger*[tw] OR northern Korea OR Pakistan*[tw] OR Papua New Guinea*[tw] OR Paraguay*[tw] OR Peru*[tw] OR Philippine*[tw] OR Principe*[tw] OR Republic of korea*[tw] OR Russia*[tw] OR Rwanda*[tw] OR Samoa*[tw] OR Serbia*[tw] OR Sao Tome*[tw] OR Senegal*[tw] OR Sierra Leone*[tw] OR Solomon Islands*[tw] OR Somalia*[tw] OR South Africa*[tw] OR “St. Lucia”[tw] OR “St. Vincent and the Grenadines”[tw] OR Surinam*[tw] OR South Sudan*[tw] OR Southern Africa*[tw] OR Sri Lanka*[tw] OR sub Saharan Africa*[tw] OR subSaharan Africa*[tw] OR sub-Saharan Africa*[tw] OR Sudan*[tw] OR Swaziland*[tw] OR Eswatini*[tw] OR Syria*[tw] OR Tajikist*[tw] OR Tanzan*[tw] OR Thai*[tw] OR Timor*[tw] OR Togo*[tw] OR Tonga*[tw] OR Tunis*[tw] OR Turk*[tw] OR Tuvalu*[tw] OR Ugand*[tw] OR Ukrain*[tw] OR Uzbekistan*[tw] OR Vanuatu*[tw] OR Venezuel*[tw] OR Vietnam*[tw] OR West Africa*[tw] OR West Bank*[tw] OR Western Africa*[tw] OR Yemen*[tw] OR Zaire*[tw] OR Zambia*[tw] OR Zimbabw*[tw] OR south east asia*[ad] OR middle east*[ad] OR

Afghan*[ad] OR Angola*[ad] OR Algeria*[ad] OR Albania*[ad] OR Armenia*[ad] OR Argentina*[ad] OR Azerbaijan*[ad] OR Bangladesh*[ad] OR Belarus*[ad] OR Benin*[ad] OR Bhutan*[ad] OR birma*[ad] OR Boliv*[ad] OR Botswan*[ad] OR Bosnia*[ad] OR Brazil*[ad] OR Bulgaria*[ad] OR Burkina Faso*[ad] OR Burundi*[ad] OR Cabo Verde*[ad] OR Cambod*[ad] OR Cameroon*[ad] OR Cape Verd*[ad] OR Central Africa*[ad] OR Chad*[ad] OR China*[ad] OR Chinese*[ad] OR Colombia*[ad] OR Comoro*[ad] OR Congo*[ad] OR Costa Rica*[ad] OR Cote d'Ivoire*[ad] OR Cuba*[ad] OR Dominica*[ad] OR Djibouti*[ad] OR East Africa*[ad] OR Eastern Africa*[ad] OR Ecuador*[ad] OR Egypt*[ad] OR El Salvador*[ad] OR Equatorial Guinea*[ad] OR Eritre*[ad] OR Ethiopia*[ad] OR Gabon*[ad] OR Gambia*[ad] OR Gaza*[ad] OR Ghan*[ad] OR Georgia*[ad] OR Grenada*[ad] OR Guatemal*[ad] OR Guinea*[ad] OR Guyana*[ad] OR Haiti*[ad] OR Hondur*[ad] OR India*[ad] OR Indones*[ad] OR Iran*[ad] OR Iraq*[ad] OR Jamaica*[ad] OR Jordan*[ad] OR Ivory Coast*[ad] OR Kazakhstan*[ad] OR Kenya*[ad] OR Kiribati*[ad] OR Kosovo*[ad] OR Kyrgyz*[ad] OR Lao PDR*[ad] OR Lesotho*[ad] OR Lebanon*[ad] OR Libya*[ad] OR Liberia*[ad] OR Macedonia*[ad] OR Madagascar*[ad] OR Malaw*[ad] OR Mali[ad] OR Mauritan*[ad] OR Malaysia*[ad] OR Maldive*[ad] OR Marshall*[ad] OR Micronesi*[ad] OR Mexic*[ad] OR Moldov*[ad] OR Mongolia*[ad] OR Montenegr*[ad] OR Morocc*[ad] OR Mozambiqu*[ad] OR Myanmar*[ad] OR Namibia*[ad] OR Nepal*[ad] OR Nicaragua*[ad] OR Niger*[ad] OR northern Korea OR Pakistan*[ad] OR Papua New Guinea*[ad] OR Paraguay*[ad] OR Peru*[ad] OR Philippine*[ad] OR Principe*[ad] OR Republic of korea*[ad] OR Russia*[ad] OR Rwanda*[ad] OR Samoa*[ad] OR Serbia*[ad] OR Sao Tome*[ad] OR Senegal*[ad] OR Sierra Leone*[ad] OR Solomon Islands*[ad] OR Somalia*[ad] OR South Africa*[ad] OR “St. Lucia”[ad] OR “St. Vincent and the Grenadines”[ad] OR Surinam*[ad] OR South Sudan*[ad] OR Southern Africa*[ad] OR Sri Lanka*[ad] OR sub Saharan Africa*[ad] OR subSaharan Africa*[ad] OR sub-Saharan Africa*[ad] OR Sudan*[ad] OR Swaziland*[ad] OR Eswatini*[ad] OR Syria*[ad] OR Tajikist*[ad] OR Tanzan*[ad] OR Thai*[ad] OR Timor*[ad] OR Togo*[ad] OR Tonga*[ad] OR Tunis*[ad] OR Turk*[ad] OR Tuvalu*[ad] OR Ugand*[ad] OR Ukrain*[ad] OR Uzbekistan*[ad] OR Vanuatu*[ad] OR Venezuel*[ad] OR Vietnam*[ad] OR West Africa*[ad] OR West Bank*[ad] OR Western Africa*[ad] OR Yemen*[ad] OR Zaire*[ad] OR Zambia*[ad] OR Zimbabw*[ad]

#7 "physical activit*"[tiab] OR "physical activit*"[keyword]

#1 AND (#2 OR #3 OR #4 OR #5) AND #6 AND #7

Scopus

#1 TITLE-ABS-KEY ("Thematic analys*" OR "content analys*" OR "focus group" * OR ethnograph* OR ethnograf* OR etnograf* OR "field stud*" OR phenomenolog* OR narration* OR narrative OR "qualitative stud*" OR "qualitative analys*" OR "qualitative research*" OR "qualitative method*" OR multimethodolog* OR "mixed method*" OR "grounded theory" OR "audio recording*" OR "tape recording*" OR audiotape* OR ((semi-structured OR semistructured OR unstructured OR informal OR in-depth OR indepth OR face-to-face OR structured OR guide*) AND (interview* OR discussion*)))

#2 TITLE-ABS-KEY(insulin AND (resistancy OR resistance OR resistant OR sensitivity))

#3 TITLE-ABS-KEY(((diabetes OR diabetic*) AND ("non insulin depend*" OR "noninsulin depend*" OR noninsulindepend* OR "non insulindepend*" OR "maturity onset*" OR "adult onset*" OR "slow onset*")) OR dm2 OR niddm OR "dm 2" OR t2d* OR "dm type 2" OR "type 2 diabet*" OR "dm type II" OR "type two diabet*" OR "type II diabet*" OR "dm type II")

#4 TITLE-ABS-KEY("metabolic syndrome" OR "glucose intoleran*" OR "glucose toleran*" OR "glucose dysregulation" OR "igt" OR "blood glucose" OR "blood sugar" OR "plasma glucose" OR "glucose level*" OR "glucose blood" OR "fasting glucose" OR "hyperglycem*" OR "hyperglycaem*" OR "Hb A1c" OR "HbA1c" OR "Hemoglobin A1c" OR "homa-ir" OR "homa2" OR "hyperinsulin*" OR "prediabet*" OR "blood pressure" OR "diastolic pressure" OR "systolic pressure" OR "pulse pressure" OR "arterial pressure" OR "aortic pressure" OR "hypertensi*" OR "triglyceride*" OR "triacylglycerol*" OR "triacetylglycerol" OR "triacetyl-glycerol" OR "trielaidin" OR "trioleoylglycerol" OR "trioleyl glycerol" OR "glycerol trioleate" OR "trioleate-glycerin" OR "hypertriglyceridemi*" OR "cholesterol" OR "hdl" OR "ldl" OR "vldl" OR "idl" OR "density lipoprotein*" OR "hdl lipoprotein*" OR "low density lipoprotein*" OR "high density lipoprotein*" OR "very low lipoprotein*" OR "obesit*" OR "obese*" OR "body mass index" OR "bmi" OR "body weight" OR "body fat*" OR "adiposit*" OR "waist hip ratio*" OR "waist to hip*" OR "waist hip ratio*" OR "waist circumference*" OR "weight change*" OR "weight gain*" OR "weight loss*" OR "weight reduction*" OR "dyslipem*" OR "dyslipidem*" OR "dyslipaem*" OR "dyslipidaem*" OR "dyslipoprotein*" OR "hyperlipemi*" OR "hyperlipid*" OR "lipidemi*" OR "lipidaemi*" OR "lipemi*"OR "hypercholesterolemi*" OR "hypercholesteremi*" OR "hyperlipoproteinemi*" OR "hypoprebetalipoproteinemi*")

#5 TITLE-ABS-KEY("cardiovascular disease*" OR cvd OR "coronary disease*" OR "heart disease*" OR atherosclerosis OR arteriosclerosis OR hypertension OR "blood pressure" OR cholesterol OR stroke* OR "peripheral vascular disease*" OR "peripheral arterial disease*")

#6 TITLE-ABS-KEY("south east asia*" OR "middle east*" OR Afghan* OR Angola* OR Algeria* OR Albania* OR Armenia* OR Argentina* OR Azerbaijan* OR Bangladesh* OR Belarus* OR Benin* OR Bhutan* OR birma* OR Boliv* OR Botswan* OR Bosnia* OR Brazil* OR Bulgaria* OR "Burkina Faso*" OR Burundi* OR "Cabo Verde*" OR Cambod* OR Cameroon* OR "Cape Verd*" OR "Central Africa*" OR Chad* OR China* OR Chinese* OR Colombia* OR Comoro* OR Congo* OR "Costa Rica*" OR "Cote d'Ivoire*"

OR Cuba* OR Dominica* OR Djibouti* OR "East Africa*" OR "Eastern Africa*" OR Ecuador* OR Egypt* OR "El Salvador*" OR "Equatorial Guinea*" OR Eritre* OR Ethiopia* OR Gabon* OR Gambia* OR Gaza* OR Ghan* OR Georgia* OR Grenada* OR Guatemal* OR Guinea* OR Guyana* OR Haiti* OR Hondur* OR India* OR Indones* OR Iran* OR Iraq* OR Jamaica* OR Jordan* OR "Ivory Coast*" OR Kazakhstan* OR Kenya* OR Kiribati* OR Kosovo* OR Kyrgyz* OR "Lao PDR*" OR Lesotho* OR Lebanon* OR Libya* OR Liberia* OR Macedonia* OR Madagascar* OR Malaw* OR Mali OR Mauritan* OR Malaysia* OR Maldive* OR Marshall* OR Micronesi* OR Mexic* OR Moldov* OR Mongolia* OR Montenegr* OR Morocc* OR Mozambiqu* OR Myanmar* OR Namibia* OR Nepal* OR Nicaragua* OR Niger* OR "northern Korea" OR Pakistan* OR "Papua New Guinea*" OR Paraguay* OR Peru* OR Philippine* OR Principe* OR "Republic of korea*" OR Russia* OR Rwanda* OR Samoa* OR Serbia* OR "Sao Tome*" OR Senegal* OR "Sierra Leone*" OR "Solomon Islands*" OR Somalia* OR "South Africa*" OR "St. Lucia" OR "St. Vincent and the Grenadines" OR Surinam* OR "South Sudan*" OR "Southern Africa*" OR "Sri Lanka*" OR "sub Saharan Africa*" OR "subSaharan Africa*" OR "sub-Saharan Africa*" OR Sudan* OR Swaziland* OR Eswatini* OR Syria* OR Tajikist* OR Tanzan* OR Thai* OR Timor* OR Togo* OR Tonga* OR Tunis* OR Turk* OR Tuvalu* OR Ugand* OR Ukrain* OR Uzbekistan* OR Vanuatu* OR Venezuel* OR Vietnam* OR "West Africa*" OR "West Bank*" OR "Western Africa*" OR Yemen* OR Zaire* OR Zambia* OR Zimbabw*) OR AFFILCOUNTRY("south east asia*" OR "middle east*" OR Afghan* OR Angola* OR Algeria* OR Albania* OR Armenia* OR Argentina* OR Azerbaijan* OR Bangladesh* OR Belarus* OR Benin* OR Bhutan* OR birma* OR Boliv* OR Botswan* OR Bosnia* OR Brazil* OR Bulgaria* OR "Burkina Faso*" OR Burundi* OR "Cabo Verde*" OR Cambod* OR Cameroon* OR "Cape Verd*" OR "Central Africa*" OR Chad* OR China* OR Chinese* OR Colombia* OR Comoro* OR Congo* OR "Costa Rica*" OR "Cote d'Ivoire*" OR Cuba* OR Dominica* OR Djibouti* OR "East Africa*" OR "Eastern Africa*" OR Ecuador* OR Egypt* OR "El Salvador*" OR "Equatorial Guinea*" OR Eritre* OR Ethiopia* OR Gabon* OR Gambia* OR Gaza* OR Ghan* OR Georgia* OR Grenada* OR Guatemal* OR Guinea* OR Guyana* OR Haiti* OR Hondur* OR India* OR Indones* OR Iran* OR Iraq* OR Jamaica* OR Jordan* OR "Ivory Coast*" OR Kazakhstan* OR Kenya* OR Kiribati* OR Kosovo* OR Kyrgyz* OR "Lao PDR*" OR Lesotho* OR Lebanon* OR Libya* OR Liberia* OR Macedonia* OR Madagascar* OR Malaw* OR Mali OR Mauritan* OR Malaysia* OR Maldive* OR Marshall* OR Micronesi* OR Mexic* OR Moldov* OR Mongolia* OR Montenegr* OR Morocc* OR Mozambiqu* OR Myanmar* OR Namibia* OR Nepal* OR Nicaragua* OR Niger* OR "northern Korea" OR Pakistan* OR "Papua New Guinea*" OR Paraguay* OR Peru* OR Philippine* OR Principe* OR "Republic of korea*" OR Russia* OR Rwanda* OR Samoa* OR Serbia* OR "Sao Tome*" OR Senegal* OR "Sierra Leone*" OR "Solomon Islands*" OR Somalia* OR "South Africa*" OR "St. Lucia" OR "St. Vincent and the Grenadines" OR Surinam* OR "South Sudan*" OR "Southern Africa*" OR "Sri Lanka*" OR "sub Saharan Africa*" OR "subSaharan Africa*" OR "sub-Saharan Africa*" OR Sudan* OR Swaziland* OR Eswatini* OR Syria* OR Tajikist* OR Tanzan* OR Thai* OR Timor* OR Togo* OR Tonga* OR Tunis* OR Turk* OR Tuvalu* OR Ugand* OR Ukrain* OR Uzbekistan* OR Vanuatu* OR Venezuel* OR Vietnam* OR "West Africa*" OR "West Bank*" OR "Western Africa*" OR Yemen* OR Zaire* OR Zambia* OR Zimbabw*)

TITLE-ABS-KEY("physical activit*")

#1 AND (#2 OR #3 OR #4 OR #5) AND #6 AND #7

Web of Science

!

#1 (TS=(("Thematic analys*" OR "content analys*" OR "focus group" * OR ethnograph* OR ethnograf* OR etnograf* OR "field stud*" OR phenomenolog* OR narration* OR narrative OR "qualitative stud*" OR "qualitative analys*" OR "qualitative research*" OR "qualitative method*" OR multimethodolog* OR "mixed method*" OR "grounded theory" OR "audio recording*" OR "tape recording*" OR audiotape* OR ((semi-structured OR semistructured OR unstructured OR informal OR in-depth OR indepth OR face-to-face OR structured OR guide*) AND (interview* OR discussion*))))) OR (AK=(("Thematic analys*" OR "content analys*" OR "focus group" * OR ethnograph* OR ethnograf* OR etnograf* OR "field stud*" OR phenomenolog* OR narration* OR narrative OR "qualitative stud*" OR "qualitative analys*" OR "qualitative research*" OR "qualitative method*" OR multimethodolog* OR "mixed method*" OR "grounded theory" OR "audio recording*" OR "tape recording*" OR audiotape* OR ((semi-structured OR semistructured OR unstructured OR informal OR in-depth OR indepth OR face-to-face OR structured OR guide*) AND (interview* OR discussion*))))) OR (AB=(("Thematic analys*" OR "content analys*" OR "focus group" * OR ethnograph* OR ethnograf* OR etnograf* OR "field stud*" OR phenomenolog* OR narration* OR narrative OR "qualitative stud*" OR "qualitative analys*" OR "qualitative research*" OR "qualitative method*" OR multimethodolog* OR "mixed method*" OR "grounded theory" OR "audio recording*" OR "tape recording*" OR audiotape* OR ((semi-structured OR semistructured OR unstructured OR informal OR in-depth OR indepth OR face-to-face OR structured OR guide*) AND (interview* OR discussion*)))))

#2 TI=(insulin AND (resistancy OR resistance OR resistant OR sensitivity)) OR AB=((insulin AND (resistancy OR resistance OR resistant OR sensitivity))) OR AK=((insulin AND (resistancy OR resistance OR resistant OR sensitivity)))

#3 TI=((((diabetes OR diabetic*) AND ("non insulin depend*" OR "noninsulin depend*" OR noninsulindepend* OR "non insulindepend*" OR "maturity onset*" OR "adult onset*" OR "slow onset*")) OR dm2 OR niddm OR "dm 2" OR t2d* OR "dm type 2" OR "type 2 diabet*" OR "dm type II" OR "type two diabet*" OR "type II diabet*" OR "dm type II")) OR AB=((((diabetes OR diabetic*) AND ("non insulin depend*" OR "noninsulin depend*" OR noninsulindepend* OR "non insulindepend*" OR "maturity onset*" OR "adult onset*" OR "slow onset*")) OR dm2 OR niddm OR "dm 2" OR t2d* OR "dm type 2" OR "type 2 diabet*" OR "dm type II" OR "type two diabet*" OR "type II diabet*" OR "dm type II")) OR AK=((((diabetes OR diabetic*) AND ("non insulin depend*" OR "noninsulin depend*" OR noninsulindepend* OR "non insulindepend*" OR "maturity onset*" OR "adult onset*" OR "slow onset*")) OR dm2 OR niddm OR "dm 2" OR t2d* OR "dm type 2" OR "type 2 diabet*" OR "dm type II" OR "type two diabet*" OR "type II diabet*" OR "dm type II"))

#4 TI=(("metabolic syndrome" OR "glucose intoleran*" OR "glucose toleran*" OR "glucose dysregulation" OR "igt" OR "blood glucose" OR "blood sugar" OR "plasma glucose" OR "glucose level*" OR "glucose blood" OR "fasting glucose" OR "hyperglycem*" OR "hyperglycaem*" OR "Hb A1c" OR "HbA1c" OR "Hemoglobin A1c" OR "homa-ir" OR "homa2" OR "hyperinsulin*" OR "prediabet*" OR "blood pressure" OR "diastolic pressure" OR "systolic pressure" OR "pulse pressure" OR "arterial pressure" OR "aortic pressure" OR "hypertensi*" OR "triglyceride*" OR "triacylglycerol*" OR "triacetylglycerol" OR "triacetyl-glycerol" OR "trielaidin" OR "trioleoylglycerol" OR "trioleyl glycerol" OR "glycerol

trioleate" OR "trioleate-glycerin" OR "hypertriglyceridemi*" OR "cholesterol" OR "hdl" OR "ldl" OR "vldl" OR "idl" OR "density lipoprotein*" OR "hdl lipoprotein*" OR "low density lipoprotein*" OR "high density lipoprotein*" OR "very low lipoprotein*" OR "obesit*" OR "obese*" OR "body mass index" OR "bmi" OR "body weight" OR "body fat*" OR "adiposit*" OR "waist hip ratio*" OR "waist to hip*" OR "waist hip ratio*" OR "waist circumference*" OR "weight change*" OR "weight gain*" OR "weight loss*" OR "weight reduction*" OR "dyslipem*" OR "dyslipidem*" OR "dyslipaem*" OR "dyslipidaem*" OR "dyslipoprotein*" OR "hyperlipemi*" OR "hyperlipid*" OR "lipidemi*" OR "lipidaemi*" OR "lipemi*"OR "hypercholesterolemi*" OR "hypercholesteremi*" OR "hyperlipoproteinemi*" OR "hypoprebetalipoproteinemi*")) OR AB=(("metabolic syndrome" OR "glucose intoleran*" OR "glucose toleran*" OR "glucose dysregulation" OR "igt" OR "blood glucose" OR "blood sugar" OR "plasma glucose" OR "glucose level*" OR "glucose blood" OR "fasting glucose" OR "hyperglycem*" OR "hyperglycaem*" OR "Hb A1c" OR "HbA1c" OR "Hemoglobin A1c" OR "homa-ir" OR "homa2" OR "hyperinsulin*" OR "prediabet*" OR "blood pressure" OR "diastolic pressure" OR "systolic pressure" OR "pulse pressure" OR "arterial pressure" OR "aortic pressure" OR "hypertensi*" OR "triglyceride*" OR "triacylglycerol*" OR "triacetylglycerol" OR "triacetyl-glycerol" OR "trielaidin" OR "trioleoylglycerol" OR "trioleyl glycerol" OR "glycerol trioleate" OR "trioleate-glycerin" OR "hypertriglyceridemi*" OR "cholesterol" OR "hdl" OR "ldl" OR "vldl" OR "idl" OR "density lipoprotein*" OR "hdl lipoprotein*" OR "low density lipoprotein*" OR "high density lipoprotein*" OR "very low lipoprotein*" OR "obesit*" OR "obese*" OR "body mass index" OR "bmi" OR "body weight" OR "body fat*" OR "adiposit*" OR "waist hip ratio*" OR "waist to hip*" OR "waist hip ratio*" OR "waist circumference*" OR "weight change*" OR "weight gain*" OR "weight loss*" OR "weight reduction*" OR "dyslipem*" OR "dyslipidem*" OR "dyslipaem*" OR "dyslipidaem*" OR "dyslipoprotein*" OR "hyperlipemi*" OR "hyperlipid*" OR "lipidemi*" OR "lipidaemi*" OR "lipemi*"OR "hypercholesterolemi*" OR "hypercholesteremi*" OR "hyperlipoproteinemi*" OR "hypoprebetalipoproteinemi*")) OR AK=(("metabolic syndrome" OR "glucose intoleran*" OR "glucose toleran*" OR "glucose dysregulation" OR "igt" OR "blood glucose" OR "blood sugar" OR "plasma glucose" OR "glucose level*" OR "glucose blood" OR "fasting glucose" OR "hyperglycem*" OR "hyperglycaem*" OR "Hb A1c" OR "HbA1c" OR "Hemoglobin A1c" OR "homa-ir" OR "homa2" OR "hyperinsulin*" OR "prediabet*" OR "blood pressure" OR "diastolic pressure" OR "systolic pressure" OR "pulse pressure" OR "arterial pressure" OR "aortic pressure" OR "hypertensi*" OR "triglyceride*" OR "triacylglycerol*" OR "triacetylglycerol" OR "triacetyl-glycerol" OR "trielaidin" OR "trioleoylglycerol" OR "trioleyl glycerol" OR "glycerol trioleate" OR "trioleate-glycerin" OR "hypertriglyceridemi*" OR "cholesterol" OR "hdl" OR "ldl" OR "vldl" OR "idl" OR "density lipoprotein*" OR "hdl lipoprotein*" OR "low density lipoprotein*" OR "high density lipoprotein*" OR "very low lipoprotein*" OR "obesit*" OR "obese*" OR "body mass index" OR "bmi" OR "body weight" OR "body fat*" OR "adiposit*" OR "waist hip ratio*" OR "waist to hip*" OR "waist hip ratio*" OR "waist circumference*" OR "weight change*" OR "weight gain*" OR "weight loss*" OR "weight reduction*" OR "dyslipem*" OR "dyslipidem*" OR "dyslipaem*" OR "dyslipidaem*" OR "dyslipoprotein*" OR "hyperlipemi*" OR "hyperlipid*" OR "lipidemi*" OR "lipidaemi*" OR "lipemi*"OR "hypercholesterolemi*" OR "hypercholesteremi*" OR "hyperlipoproteinemi*" OR "hypoprebetalipoproteinemi*"))

#5 TI=(("cardiovascular disease*" OR cvd OR "coronary disease*" OR "heart disease*" OR atherosclerosis OR arteriosclerosis OR hypertension OR "blood pressure" OR cholesterol OR stroke* OR "peripheral vascular disease*" OR "peripheral arterial disease*")) OR

AB=(("cardiovascular disease*" OR cvd OR "coronary disease*" OR "heart disease*" OR atherosclerosis OR arteriosclerosis OR hypertension OR "blood pressure" OR cholesterol OR stroke* OR "peripheral vascular disease*" OR "peripheral arterial disease*")) OR AK=(("cardiovascular disease*" OR cvd OR "coronary disease*" OR "heart disease*" OR atherosclerosis OR arteriosclerosis OR hypertension OR "blood pressure" OR cholesterol OR stroke* OR "peripheral vascular disease*" OR "peripheral arterial disease*"))

#6 TI=(("south east asia*" OR "middle east*" OR Afghan* OR Angola* OR Algeria* OR Albania* OR Armenia* OR Argentina* OR Azerbaijan* OR Bangladesh* OR Belarus* OR Benin* OR Bhutan* OR birma* OR Boliv* OR Botswan* OR Bosnia* OR Brazil* OR Bulgaria* OR "Burkina Faso*" OR Burundi* OR "Cabo Verde*" OR Cambod* OR Cameroon* OR "Cape Verd*" OR "Central Africa*" OR Chad* OR China* OR Chinese* OR Colombia* OR Comoro* OR Congo* OR "Costa Rica*" OR "Cote d'Ivoire*" OR Cuba* OR Dominica* OR Djibouti* OR "East Africa*" OR "Eastern Africa*" OR Ecuador* OR Egypt* OR "El Salvador*" OR "Equatorial Guinea*" OR Eritre* OR Ethiopia* OR Gabon* OR Gambia* OR Gaza* OR Ghan* OR Georgia* OR Grenada* OR Guatemal* OR Guinea* OR Guyana* OR Haiti* OR Hondur* OR India* OR Indones* OR Iran* OR Iraq* OR Jamaica* OR Jordan* OR "Ivory Coast*" OR Kazakhstan* OR Kenya* OR Kiribati* OR Kosovo* OR Kyrgyz* OR "Lao PDR*" OR Lesotho* OR Lebanon* OR Libya* OR Liberia* OR Macedonia* OR Madagascar* OR Malaw* OR Mali OR Mauritan* OR Malaysia* OR Maldive* OR Marshall* OR Micronesi* OR Mexic* OR Moldov* OR Mongolia* OR Montenegr* OR Morocc* OR Mozambiqu* OR Myanmar* OR Namibia* OR Nepal* OR Nicaragua* OR Niger* OR "northern Korea" OR Pakistan* OR "Papua New Guinea*" OR Paraguay* OR Peru* OR Philippine* OR Principe* OR "Republic of korea*" OR Russia* OR Rwanda* OR Samoa* OR Serbia* OR "Sao Tome*" OR Senegal* OR "Sierra Leone*" OR "Solomon Islands*" OR Somalia* OR "South Africa*" OR "St. Lucia" OR "St. Vincent and the Grenadines" OR Surinam* OR "South Sudan*" OR "Southern Africa*" OR "Sri Lanka*" OR "sub Saharan Africa*" OR "subSaharan Africa*" OR "sub-Saharan Africa*" OR Sudan* OR Swaziland* OR Eswatini* OR Syria* OR Tajikist* OR Tanzan* OR Thai* OR Timor* OR Togo* OR Tonga* OR Tunis* OR Turk* OR Tuvalu* OR Ugand* OR Ukrain* OR Uzbekistan* OR Vanuatu* OR Venezuel* OR Vietnam* OR "West Africa*" OR "West Bank*" OR "Western Africa*" OR Yemen* OR Zaire* OR Zambia* OR Zimbabw*) OR AFFILCOUNTRY("south east asia*" OR "middle east*" OR Afghan* OR Angola* OR Algeria* OR Albania* OR Armenia* OR Argentina* OR Azerbaijan* OR Bangladesh* OR Belarus* OR Benin* OR Bhutan* OR birma* OR Boliv* OR Botswan* OR Bosnia* OR Brazil* OR Bulgaria* OR "Burkina Faso*" OR Burundi* OR "Cabo Verde*" OR Cambod* OR Cameroon* OR "Cape Verd*" OR "Central Africa*" OR Chad* OR China* OR Chinese* OR Colombia* OR Comoro* OR Congo* OR "Costa Rica*" OR "Cote d'Ivoire*" OR Cuba* OR Dominica* OR Djibouti* OR "East Africa*" OR "Eastern Africa*" OR Ecuador* OR Egypt* OR "El Salvador*" OR "Equatorial Guinea*" OR Eritre* OR Ethiopia* OR Gabon* OR Gambia* OR Gaza* OR Ghan* OR Georgia* OR Grenada* OR Guatemal* OR Guinea* OR Guyana* OR Haiti* OR Hondur* OR India* OR Indones* OR Iran* OR Iraq* OR Jamaica* OR Jordan* OR "Ivory Coast*" OR Kazakhstan* OR Kenya* OR Kiribati* OR Kosovo* OR Kyrgyz* OR "Lao PDR*" OR Lesotho* OR Lebanon* OR Libya* OR Liberia* OR Macedonia* OR Madagascar* OR Malaw* OR Mali OR Mauritan* OR Malaysia* OR Maldive* OR Marshall* OR Micronesi* OR Mexic* OR Moldov* OR Mongolia* OR Montenegr* OR Morocc* OR Mozambiqu* OR Myanmar* OR Namibia* OR Nepal* OR Nicaragua* OR Niger* OR "northern Korea" OR Pakistan* OR "Papua New Guinea*" OR Paraguay* OR Peru* OR Philippine* OR Principe* OR "Republic of korea*" OR Russia* OR Rwanda* OR Samoa* OR Serbia* OR "Sao Tome*" OR Senegal* OR

"Sierra Leone*" OR "Solomon Islands*" OR Somalia* OR "South Africa*" OR "St. Lucia" OR "St. Vincent and the Grenadines" OR Surinam* OR "South Sudan*" OR "Southern Africa*" OR "Sri Lanka*" OR "sub Saharan Africa*" OR "subSaharan Africa*" OR "sub-Saharan Africa*" OR Sudan* OR Swaziland* OR Eswatini* OR Syria* OR Tajikist* OR Tanzan* OR Thai* OR Timor* OR Togo* OR Tonga* OR Tunis* OR Turk* OR Tuvalu* OR Ugand* OR Ukrain* OR Uzbekistan* OR Vanuatu* OR Venezuel* OR Vietnam* OR "West Africa*" OR "West Bank*" OR "Western Africa*" OR Yemen* OR Zaire* OR Zambia* OR Zimbabw*)) OR AB=(("south east asia*" OR "middle east*" OR Afghan* OR Angola* OR Algeria* OR Albania* OR Armenia* OR Argentina* OR Azerbaijan* OR Bangladesh* OR Belarus* OR Benin* OR Bhutan* OR birma* OR Boliv* OR Botswan* OR Bosnia* OR Brazil* OR Bulgaria* OR "Burkina Faso*" OR Burundi* OR "Cabo Verde*" OR Cambod* OR Cameroon* OR "Cape Verd*" OR "Central Africa*" OR Chad* OR China* OR Chinese* OR Colombia* OR Comoro* OR Congo* OR "Costa Rica*" OR "Cote d'Ivoire*" OR Cuba* OR Dominica* OR Djibouti* OR "East Africa*" OR "Eastern Africa*" OR Ecuador* OR Egypt* OR "El Salvador*" OR "Equatorial Guinea*" OR Eritre* OR Ethiopia* OR Gabon* OR Gambia* OR Gaza* OR Ghan* OR Georgia* OR Grenada* OR Guatemal* OR Guinea* OR Guyana* OR Haiti* OR Hondur* OR India* OR Indones* OR Iran* OR Iraq* OR Jamaica* OR Jordan* OR "Ivory Coast*" OR Kazakhstan* OR Kenya* OR Kiribati* OR Kosovo* OR Kyrgyz* OR "Lao PDR*" OR Lesotho* OR Lebanon* OR Libya* OR Liberia* OR Macedonia* OR Madagascar* OR Malaw* OR Mali OR Mauritan* OR Malaysia* OR Maldive* OR Marshall* OR Micronesi* OR Mexic* OR Moldov* OR Mongolia* OR Montenegr* OR Morocc* OR Mozambiqu* OR Myanmar* OR Namibia* OR Nepal* OR Nicaragua* OR Niger* OR "northern Korea" OR Pakistan* OR "Papua New Guinea*" OR Paraguay* OR Peru* OR Philippine* OR Principe* OR "Republic of korea*" OR Russia* OR Rwanda* OR Samoa* OR Serbia* OR "Sao Tome*" OR Senegal* OR "Sierra Leone*" OR "Solomon Islands*" OR Somalia* OR "South Africa*" OR "St. Lucia" OR "St. Vincent and the Grenadines" OR Surinam* OR "South Sudan*" OR "Southern Africa*" OR "Sri Lanka*" OR "sub Saharan Africa*" OR "subSaharan Africa*" OR "sub-Saharan Africa*" OR Sudan* OR Swaziland* OR Eswatini* OR Syria* OR Tajikist* OR Tanzan* OR Thai* OR Timor* OR Togo* OR Tonga* OR Tunis* OR Turk* OR Tuvalu* OR Ugand* OR Ukrain* OR Uzbekistan* OR Vanuatu* OR Venezuel* OR Vietnam* OR "West Africa*" OR "West Bank*" OR "Western Africa*" OR Yemen* OR Zaire* OR Zambia* OR Zimbabw*) OR AFFILCOUNTRY("south east asia*" OR "middle east*" OR Afghan* OR Angola* OR Algeria* OR Albania* OR Armenia* OR Argentina* OR Azerbaijan* OR Bangladesh* OR Belarus* OR Benin* OR Bhutan* OR birma* OR Boliv* OR Botswan* OR Bosnia* OR Brazil* OR Bulgaria* OR "Burkina Faso*" OR Burundi* OR "Cabo Verde*" OR Cambod* OR Cameroon* OR "Cape Verd*" OR "Central Africa*" OR Chad* OR China* OR Chinese* OR Colombia* OR Comoro* OR Congo* OR "Costa Rica*" OR "Cote d'Ivoire*" OR Cuba* OR Dominica* OR Djibouti* OR "East Africa*" OR "Eastern Africa*" OR Ecuador* OR Egypt* OR "El Salvador*" OR "Equatorial Guinea*" OR Eritre* OR Ethiopia* OR Gabon* OR Gambia* OR Gaza* OR Ghan* OR Georgia* OR Grenada* OR Guatemal* OR Guinea* OR Guyana* OR Haiti* OR Hondur* OR India* OR Indones* OR Iran* OR Iraq* OR Jamaica* OR Jordan* OR "Ivory Coast*" OR Kazakhstan* OR Kenya* OR Kiribati* OR Kosovo* OR Kyrgyz* OR "Lao PDR*" OR Lesotho* OR Lebanon* OR Libya* OR Liberia* OR Macedonia* OR Madagascar* OR Malaw* OR Mali OR Mauritan* OR Malaysia* OR Maldive* OR Marshall* OR Micronesi* OR Mexic* OR Moldov* OR Mongolia* OR Montenegr* OR Morocc* OR Mozambiqu* OR Myanmar* OR Namibia* OR Nepal* OR Nicaragua* OR Niger* OR "northern Korea" OR Pakistan* OR "Papua New Guinea*" OR Paraguay* OR Peru* OR Philippine* OR Principe* OR "Republic of korea*" OR Russia* OR Rwanda* OR Samoa* OR Serbia* OR "Sao Tome*" OR

Senegal* OR "Sierra Leone*" OR "Solomon Islands*" OR Somalia* OR "South Africa*" OR "St. Lucia" OR "St. Vincent and the Grenadines" OR Surinam* OR "South Sudan*" OR "Southern Africa*" OR "Sri Lanka*" OR "sub Saharan Africa*" OR "subSaharan Africa*" OR "sub-Saharan Africa*" OR Sudan* OR Swaziland* OR Eswatini* OR Syria* OR Tajikist* OR Tanzan* OR Thai* OR Timor* OR Togo* OR Tonga* OR Tunis* OR Turk* OR Tuvalu* OR Ugand* OR Ukrain* OR Uzbekistan* OR Vanuatu* OR Venezuel* OR Vietnam* OR "West Africa*" OR "West Bank*" OR "Western Africa*" OR Yemen* OR Zaire* OR Zambia* OR Zimbabw*)) OR AK=(("south east asia*" OR "middle east*" OR Afghan* OR Angola* OR Algeria* OR Albania* OR Armenia* OR Argentina* OR Azerbaijan* OR Bangladesh* OR Belarus* OR Benin* OR Bhutan* OR birma* OR Boliv* OR Botswan* OR Bosnia* OR Brazil* OR Bulgaria* OR "Burkina Faso*" OR Burundi* OR "Cabo Verde*" OR Cambod* OR Cameroon* OR "Cape Verd*" OR "Central Africa*" OR Chad* OR China* OR Chinese* OR Colombia* OR Comoro* OR Congo* OR "Costa Rica*" OR "Cote d'Ivoire*" OR Cuba* OR Dominica* OR Djibouti* OR "East Africa*" OR "Eastern Africa*" OR Ecuador* OR Egypt* OR "El Salvador*" OR "Equatorial Guinea*" OR Eritre* OR Ethiopia* OR Gabon* OR Gambia* OR Gaza* OR Ghan* OR Georgia* OR Grenada* OR Guatemal* OR Guinea* OR Guyana* OR Haiti* OR Hondur* OR India* OR Indones* OR Iran* OR Iraq* OR Jamaica* OR Jordan* OR "Ivory Coast*" OR Kazakhstan* OR Kenya* OR Kiribati* OR Kosovo* OR Kyrgyz* OR "Lao PDR*" OR Lesotho* OR Lebanon* OR Libya* OR Liberia* OR Macedonia* OR Madagascar* OR Malaw* OR Mali OR Mauritan* OR Malaysia* OR Maldive* OR Marshall* OR Micronesi* OR Mexic* OR Moldov* OR Mongolia* OR Montenegr* OR Morocc* OR Mozambiqu* OR Myanmar* OR Namibia* OR Nepal* OR Nicaragua* OR Niger* OR "northern Korea" OR Pakistan* OR "Papua New Guinea*" OR Paraguay* OR Peru* OR Philippine* OR Principe* OR "Republic of korea*" OR Russia* OR Rwanda* OR Samoa* OR Serbia* OR "Sao Tome*" OR Senegal* OR "Sierra Leone*" OR "Solomon Islands*" OR Somalia* OR "South Africa*" OR "St. Lucia" OR "St. Vincent and the Grenadines" OR Surinam* OR "South Sudan*" OR "Southern Africa*" OR "Sri Lanka*" OR "sub Saharan Africa*" OR "subSaharan Africa*" OR "sub-Saharan Africa*" OR Sudan* OR Swaziland* OR Eswatini* OR Syria* OR Tajikist* OR Tanzan* OR Thai* OR Timor* OR Togo* OR Tonga* OR Tunis* OR Turk* OR Tuvalu* OR Ugand* OR Ukrain* OR Uzbekistan* OR Vanuatu* OR Venezuel* OR Vietnam* OR "West Africa*" OR "West Bank*" OR "Western Africa*" OR Yemen* OR Zaire* OR Zambia* OR Zimbabw*) OR AFFILCOUNTRY("south east asia*" OR "middle east*" OR Afghan* OR Angola* OR Algeria* OR Albania* OR Armenia* OR Argentina* OR Azerbaijan* OR Bangladesh* OR Belarus* OR Benin* OR Bhutan* OR birma* OR Boliv* OR Botswan* OR Bosnia* OR Brazil* OR Bulgaria* OR "Burkina Faso*" OR Burundi* OR "Cabo Verde*" OR Cambod* OR Cameroon* OR "Cape Verd*" OR "Central Africa*" OR Chad* OR China* OR Chinese* OR Colombia* OR Comoro* OR Congo* OR "Costa Rica*" OR "Cote d'Ivoire*" OR Cuba* OR Dominica* OR Djibouti* OR "East Africa*" OR "Eastern Africa*" OR Ecuador* OR Egypt* OR "El Salvador*" OR "Equatorial Guinea*" OR Eritre* OR Ethiopia* OR Gabon* OR Gambia* OR Gaza* OR Ghan* OR Georgia* OR Grenada* OR Guatemal* OR Guinea* OR Guyana* OR Haiti* OR Hondur* OR India* OR Indones* OR Iran* OR Iraq* OR Jamaica* OR Jordan* OR "Ivory Coast*" OR Kazakhstan* OR Kenya* OR Kiribati* OR Kosovo* OR Kyrgyz* OR "Lao PDR*" OR Lesotho* OR Lebanon* OR Libya* OR Liberia* OR Macedonia* OR Madagascar* OR Malaw* OR Mali OR Mauritan* OR Malaysia* OR Maldive* OR Marshall* OR Micronesi* OR Mexic* OR Moldov* OR Mongolia* OR Montenegr* OR Morocc* OR Mozambiqu* OR Myanmar* OR Namibia* OR Nepal* OR Nicaragua* OR Niger* OR "northern Korea" OR Pakistan* OR "Papua New Guinea*" OR Paraguay* OR Peru* OR Philippine* OR Principe* OR "Republic of korea*" OR Russia* OR Rwanda* OR Samoa* OR Serbia* OR

"Sao Tome*" OR Senegal* OR "Sierra Leone*" OR "Solomon Islands*" OR Somalia* OR "South Africa*" OR "St. Lucia" OR "St. Vincent and the Grenadines" OR Surinam* OR "South Sudan*" OR "Southern Africa*" OR "Sri Lanka*" OR "sub Saharan Africa*" OR "subSaharan Africa*" OR "sub-Saharan Africa*" OR Sudan* OR Swaziland* OR Eswatini* OR Syria* OR Tajikist* OR Tanzan* OR Thai* OR Timor* OR Togo* OR Tonga* OR Tunis* OR Turk* OR Tuvalu* OR Ugand* OR Ukrain* OR Uzbekistan* OR Vanuatu* OR Venezuel* OR Vietnam* OR "West Africa*" OR "West Bank*" OR "Western Africa*" OR Yemen* OR Zaire* OR Zambia* OR Zimbabw*))

#7 TI=("physical activit*") OR AB=("physical activit*") OR AK=("physical activit*")

#1 AND (#2 OR #3 OR #4 OR #5) AND #6 AND #7

CINAHL, AfricaWide, and SPORTdiscus (EBSCOhost)

#1 TI ("Thematic analys*" OR "content analys*" OR "focus group" * OR ethnograph* OR ethnograf* OR etnograf* OR "field stud*" OR phenomenolog* OR narration* OR narrative OR "qualitative stud*" OR "qualitative analys*" OR "qualitative research*" OR "qualitative method*" OR multimethodolog* OR "mixed method*" OR "grounded theory" OR "audio recording*" OR "tape recording*" OR audiotape* OR ((semi-structured OR semistructured OR unstructured OR informal OR in-depth OR indepth OR face-to-face OR structured OR guide*) AND (interview* OR discussion*))) OR AB ("Thematic analys*" OR "content analys*" OR "focus group" * OR ethnograph* OR ethnograf* OR etnograf* OR "field stud*" OR phenomenolog* OR narration* OR narrative OR "qualitative stud*" OR "qualitative analys*" OR "qualitative research*" OR "qualitative method*" OR multimethodolog* OR "mixed method*" OR "grounded theory" OR "audio recording*" OR "tape recording*" OR audiotape* OR ((semi-structured OR semistructured OR unstructured OR informal OR in-depth OR indepth OR face-to-face OR structured OR guide*) AND (interview* OR discussion*))) OR SU ("Thematic analys*" OR "content analys*" OR "focus group" * OR ethnograph* OR ethnograf* OR etnograf* OR "field stud*" OR phenomenolog* OR narration* OR narrative OR "qualitative stud*" OR "qualitative analys*" OR "qualitative research*" OR "qualitative method*" OR multimethodolog* OR "mixed method*" OR "grounded theory" OR "audio recording*" OR "tape recording*" OR audiotape* OR ((semi-structured OR semistructured OR unstructured OR informal OR in-depth OR indepth OR face-to-face OR structured OR guide*) AND (interview* OR discussion*)))

#2 TI (insulin AND (resistancy OR resistance OR resistant OR sensitivity)) OR AB (insulin AND (resistancy OR resistance OR resistant OR sensitivity)) OR SU (insulin AND (resistancy OR resistance OR resistant OR sensitivity))

#3 TI ((((diabetes OR diabetic*) AND ("non insulin depend*" OR "noninsulin depend*" OR noninsulindepend* OR "non insulindepend*" OR "maturity onset*" OR "adult onset*" OR "slow onset*")) OR dm2 OR niddm OR "dm 2" OR t2d* OR "dm type 2" OR "type 2 diabet*" OR "dm type II" OR "type two diabet*" OR "type II diabet*" OR "dm type II")) OR AB ((((diabetes OR diabetic*) AND ("non insulin depend*" OR "noninsulin depend*" OR noninsulindepend* OR "non insulindepend*" OR "maturity onset*" OR "adult onset*" OR "slow onset*")) OR dm2 OR niddm OR "dm 2" OR t2d* OR "dm type 2" OR "type 2 diabet*" OR "dm type II" OR "type two diabet*" OR "type II diabet*" OR "dm type II")) OR SU ((((diabetes OR diabetic*) AND ("non insulin depend*" OR "noninsulin depend*" OR noninsulindepend* OR "non insulindepend*" OR "maturity onset*" OR "adult onset*" OR "slow onset*")) OR dm2 OR niddm OR "dm 2" OR t2d* OR "dm type 2" OR "type 2 diabet*" OR "dm type II" OR "type two diabet*" OR "type II diabet*" OR "dm type II"))

#4 TI (("metabolic syndrome" OR "glucose intoleran*" OR "glucose toleran*" OR "glucose dysregulation" OR "igt" OR "blood glucose" OR "blood sugar" OR "plasma glucose" OR "glucose level*" OR "glucose blood" OR "fasting glucose" OR "hyperglycem*" OR "hyperglycaem*" OR "Hb A1c" OR "HbA1c" OR "Hemoglobin A1c" OR "homa-ir" OR "homa2" OR "hyperinsulin*" OR "prediabet*" OR "blood pressure" OR "diastolic pressure" OR "systolic pressure" OR "pulse pressure" OR "arterial pressure" OR "aortic pressure" OR "hypertensi*" OR "triglyceride*" OR "triacylglycerol*" OR "triacetylglycerol" OR "triacetyl-glycerol" OR "trielaidin" OR "trioleoylglycerol" OR "trioleyl glycerol" OR "glycerol trioleate" OR "trioleate-glycerin" OR "hypertriglyceridemi*" OR "cholesterol" OR "hdl" OR

"ldl" OR "vldl" OR "idl" OR "density lipoprotein*" OR "hdl lipoprotein*" OR "low density lipoprotein*" OR "high density lipoprotein*" OR "very low lipoprotein*" OR "obesit*" OR "obese*" OR "body mass index" OR "bmi" OR "body weight" OR "body fat*" OR "adiposit*" OR "waist hip ratio*" OR "waist to hip*" OR "waist hip ratio*" OR "waist circumference*" OR "weight change*" OR "weight gain*" OR "weight loss*" OR "weight reduction*" OR "dyslipem*" OR "dyslipidem*" OR "dyslipaem*" OR "dyslipidaem*" OR "dyslipoprotein*" OR "hyperlipemi*" OR "hyperlipid*" OR "lipidemi*" OR "lipidaemi*" OR "lipemi*"OR "hypercholesterolemi*" OR "hypercholesteremi*" OR "hyperlipoproteinemi*" OR "hypoprebetalipoproteinemi*")) OR AB (("metabolic syndrome" OR "glucose intoleran*" OR "glucose toleran*" OR "glucose dysregulation" OR "igt" OR "blood glucose" OR "blood sugar" OR "plasma glucose" OR "glucose level*" OR "glucose blood" OR "fasting glucose" OR "hyperglycem*" OR "hyperglycaem*" OR "Hb A1c" OR "HbA1c" OR "Hemoglobin A1c" OR "homa-ir" OR "homa2" OR "hyperinsulin*" OR "prediabet*" OR "blood pressure" OR "diastolic pressure" OR "systolic pressure" OR "pulse pressure" OR "arterial pressure" OR "aortic pressure" OR "hypertensi*" OR "triglyceride*" OR "triacylglycerol*" OR "triacetylglycerol" OR "triacetyl-glycerol" OR "trielaidin" OR "trioleoylglycerol" OR "trioleyl glycerol" OR "glycerol trioleate" OR "trioleate-glycerin" OR "hypertriglyceridemi*" OR "cholesterol" OR "hdl" OR "ldl" OR "vldl" OR "idl" OR "density lipoprotein*" OR "hdl lipoprotein*" OR "low density lipoprotein*" OR "high density lipoprotein*" OR "very low lipoprotein*" OR "obesit*" OR "obese*" OR "body mass index" OR "bmi" OR "body weight" OR "body fat*" OR "adiposit*" OR "waist hip ratio*" OR "waist to hip*" OR "waist hip ratio*" OR "waist circumference*" OR "weight change*" OR "weight gain*" OR "weight loss*" OR "weight reduction*" OR "dyslipem*" OR "dyslipidem*" OR "dyslipaem*" OR "dyslipidaem*" OR "dyslipoprotein*" OR "hyperlipemi*" OR "hyperlipid*" OR "lipidemi*" OR "lipidaemi*" OR "lipemi*"OR "hypercholesterolemi*" OR "hypercholesteremi*" OR "hyperlipoproteinemi*" OR "hypoprebetalipoproteinemi*")) OR SU (("metabolic syndrome" OR "glucose intoleran*" OR "glucose toleran*" OR "glucose dysregulation" OR "igt" OR "blood glucose" OR "blood sugar" OR "plasma glucose" OR "glucose level*" OR "glucose blood" OR "fasting glucose" OR "hyperglycem*" OR "hyperglycaem*" OR "Hb A1c" OR "HbA1c" OR "Hemoglobin A1c" OR "homa-ir" OR "homa2" OR "hyperinsulin*" OR "prediabet*" OR "blood pressure" OR "diastolic pressure" OR "systolic pressure" OR "pulse pressure" OR "arterial pressure" OR "aortic pressure" OR "hypertensi*" OR "triglyceride*" OR "triacylglycerol*" OR "triacetylglycerol" OR "triacetyl-glycerol" OR "trielaidin" OR "trioleoylglycerol" OR "trioleyl glycerol" OR "glycerol trioleate" OR "trioleate-glycerin" OR "hypertriglyceridemi*" OR "cholesterol" OR "hdl" OR "ldl" OR "vldl" OR "idl" OR "density lipoprotein*" OR "hdl lipoprotein*" OR "low density lipoprotein*" OR "high density lipoprotein*" OR "very low lipoprotein*" OR "obesit*" OR "obese*" OR "body mass index" OR "bmi" OR "body weight" OR "body fat*" OR "adiposit*" OR "waist hip ratio*" OR "waist to hip*" OR "waist hip ratio*" OR "waist circumference*" OR "weight change*" OR "weight gain*" OR "weight loss*" OR "weight reduction*" OR "dyslipem*" OR "dyslipidem*" OR "dyslipaem*" OR "dyslipidaem*" OR "dyslipoprotein*" OR "hyperlipemi*" OR "hyperlipid*" OR "lipidemi*" OR "lipidaemi*" OR "lipemi*"OR "hypercholesterolemi*" OR "hypercholesteremi*" OR "hyperlipoproteinemi*" OR "hypoprebetalipoproteinemi*"))

#5 TI (("cardiovascular disease*" OR cvd OR "coronary disease*" OR "heart disease*" OR atherosclerosis OR arteriosclerosis OR hypertension OR "blood pressure" OR cholesterol OR stroke* OR "peripheral vascular disease*" OR "peripheral arterial disease*")) OR AB (("cardiovascular disease*" OR cvd OR "coronary disease*" OR "heart disease*" OR

atherosclerosis OR arteriosclerosis OR hypertension OR "blood pressure" OR cholesterol OR stroke* OR "peripheral vascular disease*" OR "peripheral arterial disease*")) OR SU (("cardiovascular disease*" OR cvd OR "coronary disease*" OR "heart disease*" OR atherosclerosis OR arteriosclerosis OR hypertension OR "blood pressure" OR cholesterol OR stroke* OR "peripheral vascular disease*" OR "peripheral arterial disease*"))

#6 TI (("south east asia*" OR "middle east*" OR Afghan* OR Angola* OR Algeria* OR Albania* OR Armenia* OR Argentina* OR Azerbaijan* OR Bangladesh* OR Belarus* OR Benin* OR Bhutan* OR birma* OR Boliv* OR Botswan* OR Bosnia* OR Brazil* OR Bulgaria* OR "Burkina Faso*" OR Burundi* OR "Cabo Verde*" OR Cambod* OR Cameroon* OR "Cape Verd*" OR "Central Africa*" OR Chad* OR China* OR Chinese* OR Colombia* OR Comoro* OR Congo* OR "Costa Rica*" OR "Cote d'Ivoire*" OR Cuba* OR Dominica* OR Djibouti* OR "East Africa*" OR "Eastern Africa*" OR Ecuador* OR Egypt* OR "El Salvador*" OR "Equatorial Guinea*" OR Eritre* OR Ethiopia* OR Gabon* OR Gambia* OR Gaza* OR Ghan* OR Georgia* OR Grenada* OR Guatemal* OR Guinea* OR Guyana* OR Haiti* OR Hondur* OR India* OR Indones* OR Iran* OR Iraq* OR Jamaica* OR Jordan* OR "Ivory Coast*" OR Kazakhstan* OR Kenya* OR Kiribati* OR Kosovo* OR Kyrgyz* OR "Lao PDR*" OR Lesotho* OR Lebanon* OR Libya* OR Liberia* OR Macedonia* OR Madagascar* OR Malaw* OR Mali OR Mauritan* OR Malaysia* OR Maldive* OR Marshall* OR Micronesi* OR Mexic* OR Moldov* OR Mongolia* OR Montenegr* OR Morocc* OR Mozambiqu* OR Myanmar* OR Namibia* OR Nepal* OR Nicaragua* OR Niger* OR "northern Korea" OR Pakistan* OR "Papua New Guinea*" OR Paraguay* OR Peru* OR Philippine* OR Principe* OR "Republic of korea*" OR Russia* OR Rwanda* OR Samoa* OR Serbia* OR "Sao Tome*" OR Senegal* OR "Sierra Leone*" OR "Solomon Islands*" OR Somalia* OR "South Africa*" OR "St. Lucia" OR "St. Vincent and the Grenadines" OR Surinam* OR "South Sudan*" OR "Southern Africa*" OR "Sri Lanka*" OR "sub Saharan Africa*" OR "subSaharan Africa*" OR "sub-Saharan Africa*" OR Sudan* OR Swaziland* OR Eswatini* OR Syria* OR Tajikist* OR Tanzan* OR Thai* OR Timor* OR Togo* OR Tonga* OR Tunis* OR Turk* OR Tuvalu* OR Ugand* OR Ukrain* OR Uzbekistan* OR Vanuatu* OR Venezuel* OR Vietnam* OR "West Africa*" OR "West Bank*" OR "Western Africa*" OR Yemen* OR Zaire* OR Zambia* OR Zimbabw*)) OR AB (("south east asia*" OR "middle east*" OR Afghan* OR Angola* OR Algeria* OR Albania* OR Armenia* OR Argentina* OR Azerbaijan* OR Bangladesh* OR Belarus* OR Benin* OR Bhutan* OR birma* OR Boliv* OR Botswan* OR Bosnia* OR Brazil* OR Bulgaria* OR "Burkina Faso*" OR Burundi* OR "Cabo Verde*" OR Cambod* OR Cameroon* OR "Cape Verd*" OR "Central Africa*" OR Chad* OR China* OR Chinese* OR Colombia* OR Comoro* OR Congo* OR "Costa Rica*" OR "Cote d'Ivoire*" OR Cuba* OR Dominica* OR Djibouti* OR "East Africa*" OR "Eastern Africa*" OR Ecuador* OR Egypt* OR "El Salvador*" OR "Equatorial Guinea*" OR Eritre* OR Ethiopia* OR Gabon* OR Gambia* OR Gaza* OR Ghan* OR Georgia* OR Grenada* OR Guatemal* OR Guinea* OR Guyana* OR Haiti* OR Hondur* OR India* OR Indones* OR Iran* OR Iraq* OR Jamaica* OR Jordan* OR "Ivory Coast*" OR Kazakhstan* OR Kenya* OR Kiribati* OR Kosovo* OR Kyrgyz* OR "Lao PDR*" OR Lesotho* OR Lebanon* OR Libya* OR Liberia* OR Macedonia* OR Madagascar* OR Malaw* OR Mali OR Mauritan* OR Malaysia* OR Maldive* OR Marshall* OR Micronesi* OR Mexic* OR Moldov* OR Mongolia* OR Montenegr* OR Morocc* OR Mozambiqu* OR Myanmar* OR Namibia* OR Nepal* OR Nicaragua* OR Niger* OR "northern Korea" OR Pakistan* OR "Papua New Guinea*" OR Paraguay* OR Peru* OR Philippine* OR Principe* OR "Republic of korea*" OR Russia* OR Rwanda* OR Samoa* OR Serbia* OR "Sao Tome*" OR Senegal* OR "Sierra Leone*" OR "Solomon Islands*" OR Somalia* OR "South Africa*" OR "St. Lucia" OR "St. Vincent

and the Grenadines" OR Surinam* OR "South Sudan*" OR "Southern Africa*" OR "Sri Lanka*" OR "sub Saharan Africa*" OR "subSaharan Africa*" OR "sub-Saharan Africa*" OR Sudan* OR Swaziland* OR Eswatini* OR Syria* OR Tajikist* OR Tanzan* OR Thai* OR Timor* OR Togo* OR Tonga* OR Tunis* OR Turk* OR Tuvalu* OR Ugand* OR Ukrain* OR Uzbekistan* OR Vanuatu* OR Venezuel* OR Vietnam* OR "West Africa*" OR "West Bank*" OR "Western Africa*" OR Yemen* OR Zaire* OR Zambia* OR Zimbabw*)) OR SU (("south east asia*" OR "middle east*" OR Afghan* OR Angola* OR Algeria* OR Albania* OR Armenia* OR Argentina* OR Azerbaijan* OR Bangladesh* OR Belarus* OR Benin* OR Bhutan* OR birma* OR Boliv* OR Botswan* OR Bosnia* OR Brazil* OR Bulgaria* OR "Burkina Faso*" OR Burundi* OR "Cabo Verde*" OR Cambod* OR Cameroon* OR "Cape Verd*" OR "Central Africa*" OR Chad* OR China* OR Chinese* OR Colombia* OR Comoro* OR Congo* OR "Costa Rica*" OR "Cote d'Ivoire*" OR Cuba* OR Dominica* OR Djibouti* OR "East Africa*" OR "Eastern Africa*" OR Ecuador* OR Egypt* OR "El Salvador*" OR "Equatorial Guinea*" OR Eritre* OR Ethiopia* OR Gabon* OR Gambia* OR Gaza* OR Ghan* OR Georgia* OR Grenada* OR Guatemal* OR Guinea* OR Guyana* OR Haiti* OR Hondur* OR India* OR Indones* OR Iran* OR Iraq* OR Jamaica* OR Jordan* OR "Ivory Coast*" OR Kazakhstan* OR Kenya* OR Kiribati* OR Kosovo* OR Kyrgyz* OR "Lao PDR*" OR Lesotho* OR Lebanon* OR Libya* OR Liberia* OR Macedonia* OR Madagascar* OR Malaw* OR Mali OR Mauritan* OR Malaysia* OR Maldive* OR Marshall* OR Micronesi* OR Mexic* OR Moldov* OR Mongolia* OR Montenegr* OR Morocc* OR Mozambiqu* OR Myanmar* OR Namibia* OR Nepal* OR Nicaragua* OR Niger* OR "northern Korea" OR Pakistan* OR "Papua New Guinea*" OR Paraguay* OR Peru* OR Philippine* OR Principe* OR "Republic of korea*" OR Russia* OR Rwanda* OR Samoa* OR Serbia* OR "Sao Tome*" OR Senegal* OR "Sierra Leone*" OR "Solomon Islands*" OR Somalia* OR "South Africa*" OR "St. Lucia" OR "St. Vincent and the Grenadines" OR Surinam* OR "South Sudan*" OR "Southern Africa*" OR "Sri Lanka*" OR "sub Saharan Africa*" OR "subSaharan Africa*" OR "sub-Saharan Africa*" OR Sudan* OR Swaziland* OR Eswatini* OR Syria* OR Tajikist* OR Tanzan* OR Thai* OR Timor* OR Togo* OR Tonga* OR Tunis* OR Turk* OR Tuvalu* OR Ugand* OR Ukrain* OR Uzbekistan* OR Vanuatu* OR Venezuel* OR Vietnam* OR "West Africa*" OR "West Bank*" OR "Western Africa*" OR Yemen* OR Zaire* OR Zambia* OR Zimbabw*))

#7 TI ("physical activit*") OR AB ("physical activit*") OR SU ("physical activit*")

#1 AND (#2 OR #3 OR #4 OR #5) AND #6 AND #7
