# Supplementary material for: Developing a Complex Understanding of Physical Activity in Cardiometabolic Disease from Low-to-Middle-Income Countries—A Qualitative Systematic Review with Meta-Synthesis
Source: Int J Environ Res Public Health. 2021 Nov 15;18(22):11977. doi: 10.3390/ijerph182211977 (PMC8619369; doi:10.3390/ijerph182211977)
Supplement: Supplementary file 1 [file ijerph-18-11977-s001.zip › File S3 - Condensed codebook.pdf]

| Synthesized finding | Category | Description | Codes |
|---|---|---|---|
| Access | Lack of transport | Access or availability of transport in relation to accessing physical activity resources | - Lack of transport limits access to physical activity programs |
| Access | Neighbourhood walkability | A measure of how well a neighbourhood promotes active forms of transportation, such as walking. A highly walkable neighbourhood has a variety of destinations within walking distance, well-connected streets, and a mix of land uses. Walkability can increase physical activity levels across a broad population | - Muddy roads lead to risk of falling<br>- Dirty roads as a barrier to physical activity<br>- Neighbourhood roads not suitable for walking<br>- Lack of a pollution free environment |
| Access | Lack of *access to* physical activity facilities | The patient has limited access to facilities conducive to physical activity. Herewith, access is distinct from the availability of such facilities. | - Lack of access to a nearby mall or shopping environment hampers physical activity participation<br>- Long distance between home and exercise environment is obstacle for physical activity |
| Access | Proximity to physical activity facility | Physical activity facilities may be available yet are not within the proximity of the patient thereby providing a barrier beyond the level that access to these facilities may be more challenging (e.g., motivation). | - Long distance between home and exercise environment is obstacle for physical activity<br>- No fitness or gym facilities within patient proximity |
| Access | Walking as an accessible form of physical activity | Walking is an easily accessible, resource-light form of physical activity. | - Walking the dog as a form of physical activity<br>- Walking was the most common form of physical activity<br>- Walking is a free form of physical activity |
| Awareness | Awareness of the exercise types or programs available | Being aware or knowledgeable of the different types of physical activity available to the patient, either through types of physical activity in public spaces, or more formal exercise/physical activity offerings. | - Better marketing of physical activity programs<br>- Not knowing about the exercise program<br>- Ignorance about available and easy everyday exercise activities<br>- Lack of exercise programs organised at health centres |

| | | | |
|---|---|---|---|
| Awareness / Diversity in Physical Activity offerings | Variety in exercise types available | A wider variety in the types of physical activity or exercise available may promote access and affinity. | - Cycling as a type of exercise engaged in by men mainly<br>- Unfamiliarity with the range of exercise types as examples of useful exercise<br>- Greater variety of physical activity programs available<br>- Excitement because of dancing<br>- Aerobic class handled by the community is available<br>- Lack of exercise programs organised at health centres |
| Capacity | Access to personal exercise equipment | Access to personal exercise equipment like treadmills, running or walking shoes, appropriate clothing, amongst others | - Access to personal exercise equipment<br>- Making use of treadmill |
| Capacity | Lack of time due to social or family responsibilities | Time for physical activity / exercise is limited due to social or family responsibilities (e.g., childcare, looking after elders). | - Household tasks limit time available for physical activity<br>- No time for exercise due to family responsibilities (e.g., children)<br>- Social responsibilities as a social-related barrier to physical activity participation<br>- Difficult to exercise and look after family |
| Capacity | Affordability of exercise / physical activity participation | The cost of participating in physical activity or exercise, including gym membership, travel cost, amongst others. While this cost may provide a barrier, in contrast, some types of physical activity are more affordable than others including walking. | - Affordability of exercise / physical activity participation<br>- Walking is a free form of physical activity |
| Community of practice | Fear of public ridicule when exercising | A fear, real or subjective, for being looked at or receiving negative feedback when exercising in public spaces | - Feeling embarrassed or uncomfortable to engage in physical activity in a public area<br>- Ashamed to exercise at home<br>- Derogatory comments or being looked upon when exercising in public |

| | | | |
|---|---|---|---|
| Cultural or religious norms | Cultural or religious norms, values or practices limit physical activity participation | Social norms and standards that are informed by local religious or cultural practices; these may impact physical activity through community of practice, stigma, perceived value, relational hierarchy, gender inequality, amongst others. | - Cultural norms around physical activity hinders adherences to physical activity recommendations<br>- Gender-specific physical activity expectations limit physical activity for women specifically<br>- Religious beliefs inform physical activity participation<br>- Authorative principles within the family inform physical activity participation<br>- Local community-views on acceptable forms of physical activity<br>- Local views that exercise is dangerous<br>- Not wanting to lose more weight |
| Dedicated facilities | Lack of adequate physical activity related facilities | Lack of dedicated spaces, specifically aimed at physical activity type activities (e.g., gyms, sports hall) | - Lack of appropriate spaces for physical activity<br>- Lack of privacy hampers physical activity participation<br>- Availability of indoor sport infrastructure may facilitate physical activity programs and participation<br>- Dedicated spaces for physical activity may promote physical activity participation |
| Diversity in physical activity offerings | Scope of available exercise / activity programs | The variety of different exercise available | - Lack of exercise programs organised at health centres |
| Knowledge | Knowledge and awareness of medical benefits related to physical activity | The patient has the knowledge or is made aware of the medical benefits of being physically active | - Lack of information on the role of exercise / physical activity in illness control<br>- Health care professional stresses the health benefits of physical activity<br>- Awareness that exercising regularly could help in preventing complications<br>- Awareness that exercising regularly could help with controlling blood sugar<br>- Awareness that exercising regularly could help with controlling blood lipids<br>- Awareness of the importance of physical activity |

| | | | |
|---|---|---|---|
| Knowledge | Health seeking behaviour | Any action or inaction undertaken by individuals who perceive themselves to have a health problem or to be ill for the purpose of finding an appropriate remedy | - Not asking for an explanation when exercise information is not understood<br>- Pro-actively obtain information from the healthcare provider |
| Life happens | | | |
| Motivation for physical activity | Lack of motivation | A lack of motivation to be physically active or exercise | - Not motivated to participate in group activities<br>- Lack of motivation a barrier to physical activity participation<br>- Being lazy as a barrier to physical activity participation<br>- Intrinsic motivation to try control illness via physical activity |
| Natural environment | Weather | The state of the atmosphere at a particular place and time, includes heat, coldness, cloudiness, dryness, sunshine, wind, rain, etc; some weather circumstances may promote or hamper participation in physical activity through access (e.g., muddy roads) or motivation (e.g., raining), amongst others. | - Cold weather imposes a barrier to PA participation<br>- Harsh weather conditions impose a barrier for PA participation<br>- Hot weather imposes a barrier to physical activity participation<br>- Rainy weather imposes a barrier to physical activity participation |
| Natural environment | An urban environment limits physical activity | Urban areas are generally very developed, meaning there is a higher density of human structures such as houses, commercial buildings, roads, bridges, and railways. In general, most employed inhabitants of urban environments have non-agricultural jobs. In some urban areas, poverty is clustered in specific regions (e.g., township, favella). | - High prevalence of sedentary occupations in an urban environment |

| | | | |
|---|---|---|---|
| Patient-provider interaction and communication | Relational proximity and trust in health care provider | A sense of relatability with the health care provider enhances trust or perceived quality of the advice, health information or services provided | - Nurse has closer relationship to patient<br>- Expert from "our people" promotes trust<br>- Provider speaks same language promotes trust<br>- Exercise is provided by a specialist |
| Patient-provider interaction and communication | Various media used to provide physical activity information | Different ways to convey information on physical activity / exercise may be conducive in specific environments, or conducive to specific patient populations. | - Lack of CDs for exercises<br>- A lack of information is described as a barrier to physical activity without specification<br>- A pamphlet with appropriate exercise may promote physical activity<br>- Healthcare providers demonstrate exercises |
| Patient-provider interaction and communication | Value of physical activity in disease management is stressed | The benefits of physical activity in relation to the medical condition or general health, are explicitly stressed with a sense of authority or urgency. | - Drugs are considered serious, not physical activity<br>- Health centre forces participation in exercise programs<br>- Health workers are instrumental in stressing the importance of physical activity as part of disease management<br>- Healthcare providers' advice on doing regular physical activity not very important |
| Person-centred care | Lack of tertiary social support | Social support or encouragement, or the lack thereof, by persons outside of the immediate or secondary support structures; includes support from those working in healthcare or service delivery. Absence of social support may negatively affect physical activity participation | - Absence of healthcare team to support adherence to PA<br>- Lack of encouragement to do PA is a perceived barrier<br>- Social support as a psychological enabler |

| | | | |
|---|---|---|---|
| Person-centred care | Information provided lacks depth and specificity | Information resources for the patient are generic, lack a level of patient-centeredness, or are provided in a (perceived) inadequate format. The lack of specific information for the patient may hamper physical activity participation, for instance to specific comorbid conditions requiring tailored exercise advice. | - The patient has been prescribed a certain exercise program, yet is unable to adhere to that program<br>- Only some information is provided without a thorough explanation<br>- Information provided is not tailored to the patient |
| Person-centred care | Exercise or physical activity is patient-centred | Exercise is most effective when tailored to the specific needs and fitness levels of the patient. | - Information provided is not made specific to the patient's comorbidities<br>- The patient has been prescribed a certain exercise program, yet is unable to adhere to that program<br>- Information provided is not tailored to the patient |
| Person-centred care | Information provided is not consistent between sources | Information provided between resources (formal or informal) is inconsistent thereby creating confusion. | - Different recommendations were provided which led to confusion<br>- Participants had different views with regards to the amount of physical activity they should be engaged in<br>- Clear lack of uniformity in the advice given by physicians regarding the amount and type of physical activity |
| Person-centred care | Regular clinical follow-ups | Repeat follow-ups with a healthcare professional allow for realignment of physical activity programming and accountability | - Once-off physical activity advice which is not re-evaluated with time<br>- Never been asked about whether I exercise regularly<br>- Add clinical evaluations as part of exercise programming |
| Personality traits | Physical activity is part of daily routine | Being physical activity is part of the daily routine and therefore a second nature; inherent to the persons day to day life. | - Having a regular exercise schedule promotes physical activity |

| | | | |
|---|---|---|---|
| Personality traits | Coping with life and stress | Conscious effort, to solve personal and interpersonal problems, to try to master, minimize or tolerate stress and conflict. Inadequate coping with life or stress-related factors may hamper physical activity participation. | - Not wanting to exercise due to family loss<br>- Not willing or able to exercise due to the stress of life<br>- Practicing activities require self-discipline<br>- Disheartened with life moves physical activity to background |
| Personality traits | Self-discipline | Ability to control one's feelings and overcome one's weaknesses; in relation to physical activity, this may mean going to exercise even if one does not feel like it. | - Practicing activities requires self-discipline |
| Personality traits | Acceptance of illness | The lack of negative responses and emotions associated with the condition | - Making terms with the chronicity of the condition and implications on daily life |
| Personality traits | Doing exercises you like | Participating exercise or physical activity types or programs which are perceived as engaging and fun to the participant. | - Excitement because of dancing<br>- Bored with exercising<br>- Patient hates any form of exercise |
| Physical activity is a necessity | Physical activity is part of (daily) commute | People's (daily) commute from home to the workplace is either walking or cycling. | - Cycling to work fulfilled physical activity requirements<br>- Cycling as a type of exercise engaged in by men mainly<br>- Ownership of motorized transport reduces physical activity |
| Physical activity is a necessity | Adapting or promoting familiar sources of physical activity | Changing the frequency, intensity, or duration of familiar source of physical activity (e.g., household chores, walking community) may provide a feasible way to increase physical activity levels. | - Using common activities for physical activity promotes physical activity participation<br>- Most feasible form of physical activity adjustment was by increasing domestic work<br>- Physical activity is related to household chores<br>- Physical activity can be adjusted with what we already do |
| Physical well-being | Fatigue | Fatigue, either physical, mental or both, is generally difficult to describe, though words like lethargic, exhausted and tiredness are used commonly. In relation to physical activity, fatigue is perceived as a barrier. | - Bodily fatigue<br>- Lack of energy<br>- Lethargy<br>- Feeling tired from working |

| | | | |
|---|---|---|---|
| Physical well-being | Post-exercise fatigue | Experience of fatigue or exertion as a result from engaging in exercise or physical activity | - Exercise-induced fatigue is limiting |
| Physical well-being | Physical health | Physical discomfort or impairments, not specifically related to a medical condition (e.g., being overweight), which impact the ability to engage in physical activity. | - Being overweight limits ambulation and physical activity<br>- Walking with crutches hampers physical activity participation<br>- Physical health limits the frequency at which physical activity could be engaged in<br>- Difficulty ambulating hampers physical activity<br>- A long history of being overweight<br>- Physical health limits the type and frequency of physical activities feasible to engage in |
| Physical well-being | Medical conditions or comorbidity | The presence of distinct comorbid conditions and/or medical conditions (e.g., heart disease) | - Low active due to Diabetes<br>- Multimorbidity limits physical activity participation<br>- Arthrosis<br>- Heart disease<br>- Poor eyesight or visual impairment |
| Physical well-being | Physical symptoms | Specific physical symptoms (e.g., pain) limit the ability to engage in physical activity, these include pain, numb feet, and others. | - Physical health limits the type and frequency of physical activities feasible to engage in<br>- Painful feet limit physical activity<br>- Unspecific body pain restricting PA / Exercise<br>- Numbness or tingling feeling in the legs<br>- Lack of stamina restricts physical activity |

| | | | |
|---|---|---|---|
| Physical well-being | Exercise-induced symptoms or exacerbations | Symptoms or worsening of a symptom brought about by exercise; possibly more prevalent in uncontrolled cardiometabolic disease or with comorbidity. | - Dizziness in relation to physical activity<br>- Fear of exercise-induced heart attack<br>- Feeling unwell from exercise<br>- Pain during exercise<br>- Shortness of breath during physical activity / exercise resulting in reduced physical activity<br>- Arthritis<br>- Chest pain when exercise as a barrier to physical activity<br>- Bradycardia when exercising<br>- Developing high blood pressure with physical exertion<br>- High blood sugar with physical exertion |
| Physical well-being | Aging | The process of getting older; May impose physical barriers to physical activity participation yet also culturally driven norms and values with respect to physical activity participation at older age. | - Getting older limits physical activity |
| Pollution | Air pollution | A mixture of solid particles and gases in the air. Car emissions, chemicals from factories, dust, pollen, and mould spores may be suspended as particles. Smog may affect physical activity participation | - Air pollution hampers physical activity participation |
| Public facilities | Lack of physical activity public and recreational infrastructure | Lack of safe or adequate spaces for physical activity that are available to all people within a specific community (i.e., public); may include walkways, green space, etc. | - Lack of walkways dedicated to pedestrians<br>- Lack of parks / green space an impediment to exercise<br>- Lack of appropriate spaces for physical activity<br>- Lack of a pollution free environment<br>- Dedicated spaces for physical activity may promote physical activity participation |
| Public safety and violence | Public safety and violence | The level of protection from crime, disaster, or other potential dangers; has an impact on access to physical activity spaces, in particular public spaces. | - Physical activity in groups is preferred<br>- Unsafe to exercise or walk outside of their home<br>- Parks and walkways particularly unsafe for women<br>- Safety in the neighbourhood a great concern |

| | | | |
|---|---|---|---|
| Recognizing the value of physical activity | Perceived benefits of physical activity on mental health and well-being | The patient recognizes the potential benefits of being physically active on aspects of mental health and well-being. Mental health and well-being in the broader sense, includes aspects related to spirituality and feelings like "happiness" or feeling "activated". | - Perceived spiritual benefits promote physical activity<br>- Perceived benefit of feeling fit promotes physical activity<br>- Perceived benefit of feeling alert promotes physical activity<br>- Feeling good and active after physical activity promotes participation<br>- Happiness during physical activity promotes participation<br>- Perceived relation between physical activity and stress control<br>- Engaging in physical activity improves sleep |
| Recognizing the value of physical activity | Perceived benefits of physical activity on cognitive health and well-being | The patient recognizes the potential benefits of being physically active on cognitive health and well-being, including cognitive factors like concentration, and feeling alert | - Perceived benefit of feeling alert promotes physical activity<br>- Feeling good and active after physical activity promotes participation |
| Recognizing the value of physical activity | Perceived negative consequences of physical activity | Potential perceived or real negative effects related to physical activity. | - Not wanting to lose more weight |
| Recognizing the value of physical activity | Positive history with the benefits of physical activity | A positive history or feedback with the potential benefits of exercising or being physical active. | - No tangible feedback on the benefits of physical activity limit continued participation<br>- Positive outcome of exercise following past stroke<br>- Patients who obtained positive results and experiences from physical activity are encouraged to continue this behaviour |
| Recognizing the value of physical activity | Perceived benefits of physical activity on physical health and well-being | Perceived benefits of physical activity on outcomes or factors related to the physical (e.g., energy, weight) health and well-being (e.g., longevity). Perceived benefits act as potential facilitators for physical activity; or barriers in the absence thereof. | - Perception that being active makes the body healthy<br>- Physical activity promotes longevity<br>- Engaging in physical activity improves sleep<br>- Physical activity perceived important to increase energy for work<br>- Exercise helps losing weight |

| | | | |
|---|---|---|---|
| Recognizing the value of physical activity | Affinity with physical activity | Attraction to or liking for physical activity | - Social upbringing where there was no opportunity to exercise<br>- Everyone in the family is overweight<br>- Exercise alien to lifestyle<br>- Patient hates any form of exercise<br>- Healthcare providers' advice on doing regular physical activity not very important<br>- Bored with exercising<br>- Having a positive attitude towards physical activity<br>- Excitement because of dancing |
| Recognizing the value of physical activity | Negative experiences | Negative experiences with accessing or engaging in exercise or physical activities; relates to the actual participation, not the perceived negative consequences of being active. | - Negative experiences with hospital-based exercise programming |
| Self-efficacy | Fear of exercise-induced adverse events | A fear of apprehension with respect to potential adverse events that occur because of or during exercise or physical activity participation. | - Awareness of the potential health risks associated with physical activity in relation to the patients’ medical condition<br>- Developing high blood pressure with physical exertion<br>- Family members fearful of encountering emergencies<br>- Afraid that physical activity will induce chest pain<br>- Local views that exercise is dangerous<br>- Fear of exercise-induced heart attack |
| Self-efficacy | Knowledge about physical activity / exercise | Having knowledge about the role of physical activity / exercise in disease management, disease aetiology, yet also understanding the difference between exercise and physical activity. Knowledge promotes health literacy and may positively impact adequate physical activity participation. | - Knowledge about recommended physical activity requirements promotes adequate physical activity<br>- Physical activity is viewed as "informal day-to-day activities" rather than organized exercise<br>- Knowledge of physical inactivity as a risk factor for Diabetes<br>- Understanding the potential benefits of engaging in physical activity |

| | | | |
|---|---|---|---|
| Self-efficacy | Exercise self-efficacy | The belief and conviction that one can successfully perform physical activity or exercise, adequately and safely. | - Awareness of the precautions to be taken before physical activity<br>- Ceasing work to engage in a more restful form of activity to maintain health<br>- Feeling insecure about engaging in physical activities<br>- Incorporating educational opportunities with exercise programming<br>- Lack of skill to join an exercise program as a psychological barrier to physical activity<br>- Not having the skill of regular exercise programming<br>- Not being aware of practices related to warming-up and cooling down<br>- Cannot differentiate exercise from routine physical activity<br>- Awareness of the importance of physical activity<br>- Incorporating educational opportunities with exercise programming |
| Social support | Secondary social support | Social support or encouragement, or the lack thereof, from individuals just outside of the immediate social support structure, including neighbours and friends. Absence of social support may negatively affect physical activity participation | - Lack of encouragement to do PA is a perceived barrier<br>- Absence of social support from neighbours<br>- Lack or absence of sense of community to inform activities<br>- Social support as a psychological enabler<br>- Absence of exercise buddies |

| | | | |
|---|---|---|---|
| Social support | Immediate social support | Social support or encouragement, or the lack thereof, by persons in the immediate environment of the patient, including family, partner. Absence of social support may negatively affect physical activity participation | - Family-based exercise programs facilitate physical activities<br>- Inadequate family support to free up time for PA<br>- Controlling behaviour by immediate support structures limit physical activity or cause distress<br>- Indifference of family towards struggle with disease self-management<br>- Difficult to exercise and look after family<br>- Absence of exercise buddies<br>- Friends encourage physical activity participation<br>- Social support as a psychological enabler<br>- Family members provide reminders about PA |
| Social support | Negative social pressure | The social support structure is negative towards or pressing in the patient' physical activity behaviour to a level it is counterproductive or "off-putting". | - Scolding or nagging behaviours<br>- Negative attitude towards to patient engaging in physical activity |
| Socio-economic well-being | Impact on socio-economic prosperity | When physical activity is an integral part of day-to-day living, whether employment or subsistence farming, the ability to engage in physical activity may have a direct effect on financial or food security. | - Unable to participate in agricultural activities essential for survival |
| Time | Time flexibility | a schedule or arrangement that allows flexibility in terms of when to be physically active or exercise; may promote physical activity for some, while others may require more structure | - Inability to effectively manage time a barrier to engaging in physical activity<br>- Engaging in physical activity as time allows |
| Time | Lack of time due employment | People who are employed (paid or unpaid) may experience lack of time as a barrier to physical activity participation | - Employment as a barrier to engaging in physical activity<br>- Travelling for work<br>- High prevalence of sedentary occupations in an urban environment |

| | | | |
|---|---|---|---|
| Urban environment limits physical activity | Type of work | The type of employment (paid or unpaid); has a direct relationship with habitual physical activity, with manual labour and farming as prime examples of work that is physically demanding, while an employment in urban environment is generally more sedentary at times. | - Physical activity is related to manual labour<br>- Physical activity is related to farming<br>- Physical activity is related to household chores<br>- High prevalence of sedentary occupations in an urban environment |
