# Supplementary material for: Developing a Complex Understanding of Physical Activity in Cardiometabolic Disease from Low-to-Middle-Income Countries—A Qualitative Systematic Review with Meta-Synthesis
Source: Int J Environ Res Public Health. 2021 Nov 15;18(22):11977. doi: 10.3390/ijerph182211977 (PMC8619369; doi:10.3390/ijerph182211977)
Supplement: Supplementary file 1 [file ijerph-18-11977-s001.zip › File S4 - Risk of bias.pdf]

File S4: Risk of bias

Study	Item 1	Item 2	Item 3	Item 4	Item 5	Item 6	Item 7	Item 8	Item 9	Item 10
Advika et al. 2017	Yes	Yes	Yes	Yes	Yes	No	Yes	Yes	Yes	Yes
Arovah et al. 2019	Yes	Yes	Yes	Yes	Yes	No	No	Yes	Yes	Yes
Basu et al. 2020	Unclear	Unclear	Yes	Yes	Yes	No	No	Yes	Yes	No
Buksh et al. 2020	Yes	Yes	Yes	Yes	Yes	No	No	Yes	Yes	Yes
Daivadanam et al. 2013	Yes	Yes	Yes	Yes	Yes	No	No	Unclear	Yes	Yes
de Graft-Aikins et al. 2020	Yes	Yes	Yes	Yes	Yes	Unclear	No	Yes	Yes	Yes
de Lima Lopes et al. 2013	Yes	Yes	Yes	Yes	Yes	Yes	No	Yes	Yes	Yes
Gebrezgi et al. 2017	Unclear	Yes	Yes	No	Yes	No	No	Yes	Yes	Yes
Ide et al. 2018	Yes	Yes	Yes	Yes	Yes	No	No	Yes	Yes	Yes
Islam et al. 2017	Yes	Yes	Yes	Yes	Yes	No	No	Yes	Yes	Yes
Jayanna et al. 2019	Unclear	Yes	Yes	Yes	Yes	No	No	Yes	Yes	Yes
Katena et al. 2015	No	Yes	Yes	No	Yes	No	No	No	Yes	Unclear
Masupe et al. 2018	Yes	Yes	Yes	Yes	Yes	No	No	Yes	Yes	Yes
Mayega et al. 2014	Yes	Yes	Yes	Yes	Yes	Yes	Unclear	Yes	Yes	Yes
Medagama et al. 2018	Unclear	Unclear	Yes	Yes	No	No	No	No	Yes	Unclear
Mickael et al. 2019	Unclear	Yes	Yes	Yes	Yes	No	No	Yes	Yes	Yes
Mizutani et al. 2016	Unclear	Yes	Yes	Yes	Yes	Yes	No	Yes	Yes	Yes
Morrison et al. 2019	Yes	Yes	Yes	Yes	Yes	No	Yes	Yes	Yes	Yes
Mphwante et al. 2021	Unclear	Yes	Yes	Unclear	Yes	No	No	Yes	Yes	Yes
Nyaaba et al. 2019	Unclear	Yes	Yes	Yes	Yes	No	No	Yes	Yes	Yes
Oba et al. 2020	No	Unclear	Yes	Yes	Yes	No	No	Yes	Yes	Yes
Odusola et al. 2014	Yes	Yes	Yes	Yes	Yes	Yes	Yes	Yes	Yes	Yes
Oliviera et al. 2015	Unclear	Yes	Yes	Yes	No	No	No	Yes	Yes	Unclear
Oo et al. 2020	Yes	Yes	Yes	Yes	Yes	Yes	Yes	Yes	Yes	Yes
Péres et al. 2007	No	No	Yes	Unclear	Unclear	No	No	Yes	Unclear	Yes
Pesantes et al. 2018	Yes	Yes	Yes	Yes	Yes	No	No	Yes	Yes	Yes
Pesantes et al. 2020	Yes	Yes	Yes	Yes	Yes	Unclear	No	Yes	Yes	Yes
Ranasinghe et al. 2015	Yes	Yes	Yes	Yes	Yes	Unclear	Yes	Yes	Yes	Yes
Rani et al. 2019	Yes	Unclear	Yes	Yes	Yes	No	No	Yes	Yes	Yes
Resende et al. 2018	Unclear	Yes	Unclear	Yes	Yes	No	No	Yes	Yes	Yes
Sabzmakan et al. 2014	Yes	Yes	Yes	Yes	Yes	Unclear	Yes	Yes	Yes	Yes
Seiffert et al. 2014	Yes	Yes	Unclear	Yes	Unclear	No	No	Yes	Yes	Unclear
Sendhilkumar et al. 2017	Unclear	No	Yes	Unclear	No	No	No	No	Yes	Unclear
Shima et al. 2014	Yes	Yes	Yes	Yes	Yes	Unclear	No	Yes	Yes	Yes
Silva et al. 2018	Yes	Yes	Yes	Yes	Yes	Unclear	Yes	Yes	Yes	Yes
Steyl et al. 2014	Unclear	Yes	Yes	Yes	Yes	No	No	Yes	Yes	Yes
Suparee et al. 2015	Yes	Yes	Yes	Yes	Yes	Unclear	Unclear	Yes	Yes	Yes
Vafa et al. 2020	Yes	Yes	Yes	Yes	Yes	No	No	Yes	Yes	Yes
Wijesinghe et al. 2013	Yes	Yes	Yes	Yes	Yes	Yes	Yes	Yes	Yes	Yes
Zare et al. 2020	Yes	Yes	Yes	Yes	Yes	No	Unclear	Yes	Yes	Yes
Zhu et al. 2011	Yes	Yes		Yes	Yes	No	No	Yes	Yes	Yes

Item 1: Is there congruity between the stated philosophical perspective and the research methodology?
Item 2: Is there congruity between the research methodology and the research question or objectives?
Item 3: Is there congruity between the research methodology and the methods used to collect data?
Item 4: Is there congruity between the research methodology and the representation and analysis of data?
Item 5: Is there congruity between the research methodology and the interpretation of results?
Item 6: Is there a statement locating the researcher culturally or theoretically?
Item 7: Is the influence of the researcher on the research, and vice-versa, addressed?
Item 8: Are participants, and their voices, adequately represented?
Item 9: Is the research ethical according to current criteria or for recent studies, and is there evidence of approval by an appropriate body?
Item 10: Do the conclusions drawn in the research report flow from the analysis, or interpretation, of the data?
