# Supplementary figures and images for: Developing a Complex Understanding of Physical Activity in Cardiometabolic Disease from Low-to-Middle-Income Countries—A Qualitative Systematic Review with Meta-Synthesis
Source: Int J Environ Res Public Health. 2021 Nov 15;18(22):11977. doi: 10.3390/ijerph182211977 (PMC8619369; doi:10.3390/ijerph182211977)

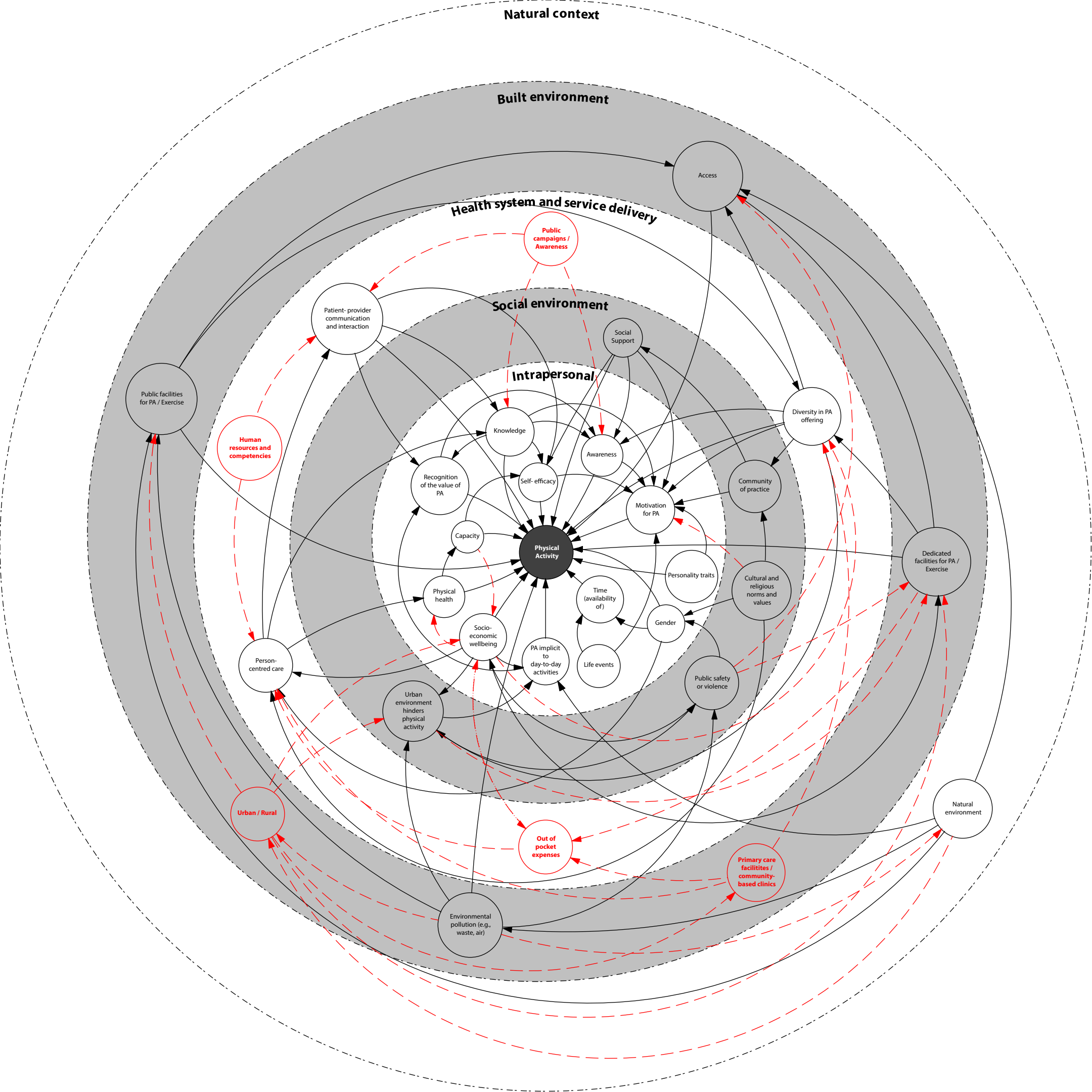

Supplement: Supplementary file 1 [file ijerph-18-11977-s001.zip › File S5- Figure 3 High Res.pdf]
